# Diazaquinomycins E–G, Novel Diaza-Anthracene Analogs from a Marine-Derived *Streptomyces* sp.

**DOI:** 10.3390/md12063574

**Published:** 2014-06-11

**Authors:** Michael W. Mullowney, Eoghainín Ó hAinmhire, Anam Shaikh, Xiaomei Wei, Urszula Tanouye, Bernard D. Santarsiero, Joanna E. Burdette, Brian T. Murphy

**Affiliations:** 1 Department of Medicinal Chemistry and Pharmacognosy, University of Illinois at Chicago, Chicago, IL 60612, USA; E-Mails: mmullo2@uic.edu (M.W.M.); eohain2@uic.edu (E.Ó.H.); ashaikh5@gmail.com (A.S.); xmchuic12@gmail.com (X.W.); urszula.tanouye@gmail.com (U.T.); bds@uic.edu (B.D.S.); joannab@uic.edu (J.E.B.); 2 Center for Pharmaceutical Biotechnology, University of Illinois at Chicago, Chicago, IL 60607, USA

**Keywords:** actinomycete, marine, *Streptomyces*, ovarian cancer, OVCAR5, diazaquinomycin

## Abstract

As part of our program to identify novel secondary metabolites that target drug-resistant ovarian cancers, a screening of our aquatic-derived actinomycete fraction library against a cisplatin-resistant ovarian cancer cell line (OVCAR5) led to the isolation of novel diaza-anthracene antibiotic diazaquinomycin E (DAQE; **1**), the isomeric mixture of diazaquinomycin F (DAQF; **2**) and diazaquinomycin G (DAQG; **3**), and known analog diazaquinomycin A (DAQA; **4**). The structures of DAQF and DAQG were solved through deconvolution of X-Ray diffraction data of their corresponding co-crystal. DAQE and DAQA exhibited moderate LC_50_ values against OVCAR5 of 9.0 and 8.8 μM, respectively. At lethal concentrations of DAQA, evidence of DNA damage was observed via induction of apoptosis through cleaved-PARP. Herein, we will discuss the isolation, structure elucidation, and biological activity of these secondary metabolites.

## 1. Introduction

Since the 1940s, natural products have proven essential as both a direct source of small molecule cancer therapies and as an inspiration for biologically active synthetic analogs of natural products; in total 131 of the 175 small molecule cancer drugs are natural products or are derived from natural products [1]. Ovarian cancer, which is the fifth leading cause of cancer death and most lethal gynecological disease in US women, would greatly benefit from new therapies [2]. New drugs are needed because all patients are treated with the same regimen of carboplatin/paclitaxel, and sadly chemoresistant disease reoccurs in most women, resulting in death [3,4].

Our program explores the capacity of actinomycete bacteria isolated from both marine and freshwater environments to produce biologically active secondary metabolites. Screening of our actinomycete secondary metabolite library against the ovarian cancer cell line OVCAR5 led to the selection of strain F001 for further chemical investigation. After several chromatographic purification steps using bioassay-guided fractionation, we isolated diazaquinomycin E (DAQE; **1**), the isomeric mixture of diazaquinomycins F and G (DAQF, **2**; DAQG, **3**), and the known analog diazaquinomycin A (DAQA; **4**). The three new compounds are mono-normethyl analogs of the diazaquinomycin structural class. DAQE and DAQA inhibited the growth of OVCAR5 cells with moderate potency (LC_50_ = 9.0 and 8.8 μM, respectively). Further biological evaluation was performed on the most abundant analog, DAQA, while we were unable to evaluate the biological activity of the isomeric mixture of DAQF and DAQG due to their low mass yield. Details of the elucidation and biological activities are described herein.

## 2. Results and Discussion

### 2.1. Structure Elucidation of DAQE, DAQF, and DAQG

**Figure 1:**
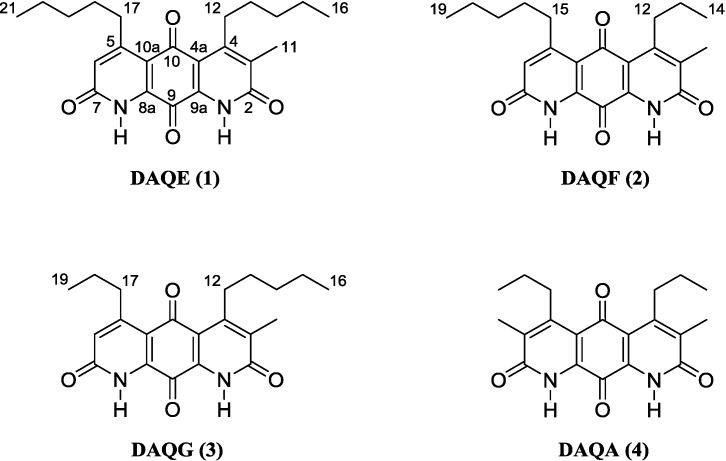
Structure of diazaquinomycins E (**1**), F (**2**), G (**3**), and A (**4**).

Following a series of chromatographic steps, **1** (Figure 1) was obtained as red powder. The molecular formula was assigned as C_23_H_28_N_2_O_4_ on the basis of combined nuclear magnetic resonance (NMR) and high-resolution mass spectrometry (MS) experiments. This formula demanded 11 degrees of unsaturation. The unique chromophore of a fused diaza-anthracene ring system consistent with the diazaquinomycin structural class was observed in the UV spectrum of **1**. Analysis of the ^1^H-NMR spectrum of **1** suggested the presence of an isolated aromatic hydrogen (δ_H_ 6.94, s, H-6). Integration of the H-6, H_3_-11, H_2_-12, H_2_-17, H_3_-16, and H_3_-21 resonances in the ^1^H spectrum further supported a mono-normethylated DAQ core skeleton, revealing an integration value of three for the α-substituted methyl hydrogens (δ_H_ 2.32, H_3_-11) rather than the typical integration value of 6 for this resonance in previously reported symmetric DAQs. This mono-normethylation afforded an asymmetry to **1**.

**Figure 2:**
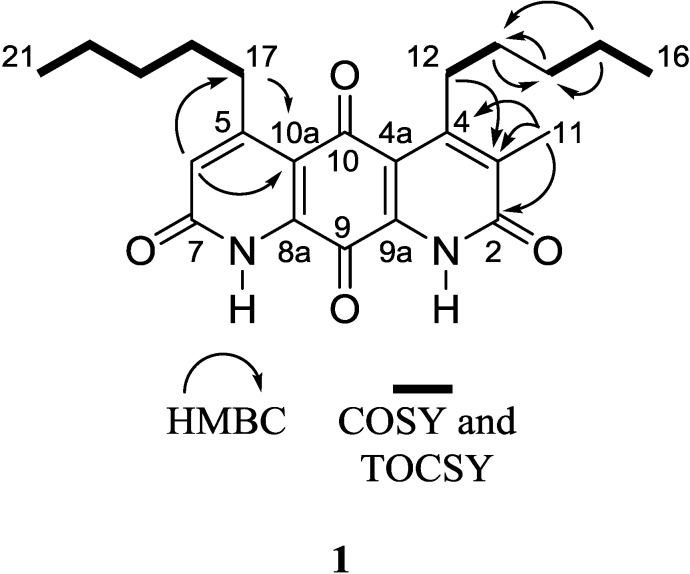
Key 2D NMR correlations of **1**.

**Figure 3:**
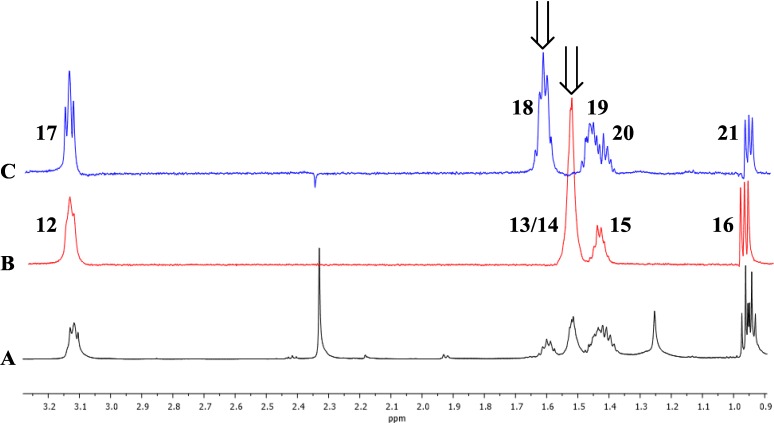
1D TOCSY correlations of **1**. Arrows indicate irradiated resonances. (**A**) Expansion of ^1^H-NMR spectrum (600 MHz) of **1**; (**B**) Expansion of 1D TOCSY spectrum of **1** (irradiation of 1.51 ppm); (**C**) Expansion of 1D TOCSY spectrum of **1** (irradiation of 1.62 ppm).

Analysis of ^13^C-NMR data suggested the presence of two quinone carbonyls (δ_C_ 180.8, C-10; 173.1, C-9), two lactam carbonyls (δ_C_ 163.0, C-2; 162.9, C-7), one methine alkene carbon (δ_C_ 127.5, C-6), seven quaternary carbons (δ_C_ 159.7, C-5; 154.2, C-4; 137.8, C-3; 136.5, C-8a; 134.1, C-9a; 118.0, C-4a; 117.8, C-10a), eight methylene carbons (δ_C_ 34.9, C-17; 32.3, C-14; 31.8, C-19; 30.7, C-12; 29.6, C-18; 28.8, C-13; 22.5, C-15; 22.5, C-20), and three methyl carbons (δ_C_ 14.1, C-16; 14.1, C-21; 13.0, C-11) (Table 1). Given that the molecular formula afforded 11 degrees of unsaturation and the molecule contained four carbonyls and eight quaternary alkene carbons, the remaining degrees were satisfied by the fused ring system. Key HMBC, COSY, and 1D-TOCSY correlations are given in Figure 2. Since the molecule is asymmetric, we were able to employ proton-based spectroscopic experiments to distinguish between resonances of the β-substituted alkyl groups. Interpretation of COSY and 1D-TOCSY data defined two distinct spin systems, which were then connected to the core ring system using HMBC correlations (Figure 3 and Supplementary Figures S4–S8).

**Table 1:** ^1^H and ^13^C-NMR data (CDCl_3_/1% CF_3_CO_2_D) of **1**.

Position	^13^C ^a^	^1^H mult. (*J*, Hz) ^b^
2	163.0 ^c^	
3	137.8	
4	154.2	
4a	118.0	
5	159.7	
6	127.5	6.94 s
7	162.9^c^	
8a	136.5	
9	173.1	
9a	134.1	
10	180.8	
10a	117.8	
11	13.0	2.32 s
12	30.7	3.12 bt (6.4)
13	28.8	1.52 m
14	32.3	1.51 m
15	22.5	1.42 m
16	14.1	0.96 t (7.3)
17	34.9	3.11 t (7.7)
18	29.6	1.60 p (7.6)
19	31.8	1.43 m
20	22.5	1.40 m
21	14.1	0.94 t (7.3)

^a^ 226.2 MHz; ^b^ 900 MHz; ^c^ resonances are interchangeable.

An HMBC correlation from H_2_-17 to C-10a, and of H-6 to C-17 and C-10a, positioned the spin system of C-17–C-18–C-19–C-20–C-21 on the normethyl half of the diaza-anthracene core. Similarly, an HMBC correlation from H_2_-12 to C-3 positioned the spin system of C-12–C-13–C-14–C-15–C-16 on the α-methylated ring of the diaza-anthracene core. Two lactam carbonyl resonances were observed in the ^13^C DEPTQ spectrum; due to overlap it was not possible to distinguish between them in an HMBC experiment (Table 1). The remaining quinone carbons C-8a, C-9, C-9a, and C-10 were assigned based on comparison with reported values of other diazaquinomycin analogs [5,6]; our assignments were highly consistent with reported values. Therefore, the structure of **1** was determined as shown and named diazaquinomycin E.

Following a series of chromatographic steps, the co-eluting isomeric mixture of **2** and **3** (Figure 1) was obtained as red powder. The molecular formula for the mixture of constitutional isomers was assigned as C_21_H_24_N_2_O_4_ on the basis of combined NMR and high-resolution MS experiments. This formula demanded 11 degrees of unsaturation. A chromophore of a fused diaza-anthracene ring system consistent with **1** was observed in the UV spectrum of the compound mixture. The diaza-anthracene core of DAQF and DAQG was determined to be mono-methylated as was previously described for **1**. This was evidenced by the presence of an aromatic hydrogen in the ^1^H-NMR spectrum (δ_H_ 6.98, s, H-6 of **2** or **3**). Evidence for a second minor isomer was observed adjacent to the resonance at δ_H_ 6.98 (δ_H_ 6.95, s, H-6 of **2** or **3**) (Supplementary Information, Figures S11 and S14). Integration of the major aromatic hydrogen and the n-pentyl and n-propyl group resonances in the ^1^H spectrum further supported a mono-normethylated DAQ core skeleton. Distinguishing separate, complete sets of ^13^C shifts from DEPTQ and HMBC experiments was not possible due to the structural similarity and inability to separate **2** and **3**. Partial carbon shift data shared by the isomers was extracted from an HSQC experiment (Supplementary Information, Table S1). Interpretation of COSY data defined two distinct spin systems, one n-pentyl group and one n-propyl group.

**Figure 4:**
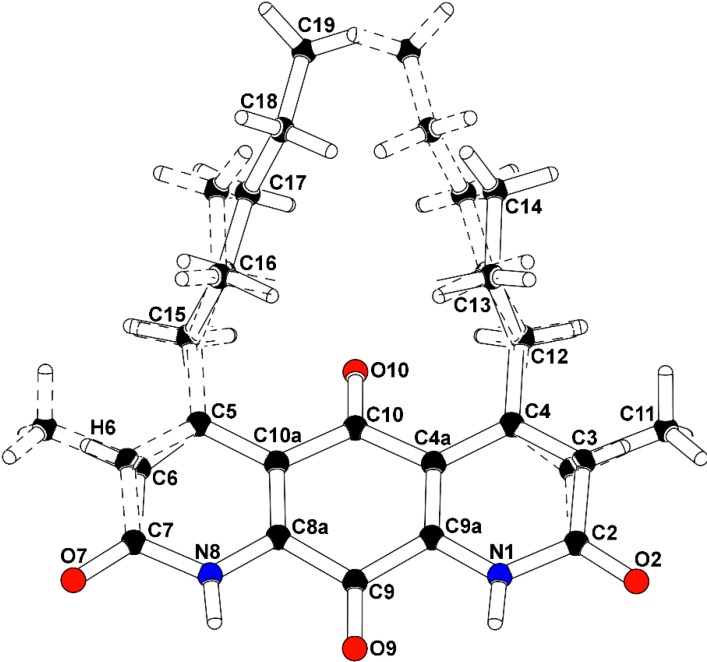
Co**-**crystal structure of diazaquinomycin F (**2**) and diazaquinomycin G (**3**).

To confirm the structural features observable by NMR analysis and to determine the remaining connectivity of the structures, an X-ray structure determination was attempted. The mixture of **2** and **3** co-crystallized from methanol using a slow evaporation technique. Compounds **2** and **3** occupied the same molecular site, crystallizing in the monoclinic space group P2_1_/m (No. 11), with a mirror plane bisecting the molecules through the central carbonyl atoms C9-O9 and C10-O10. The major isomer (**2**), present as 52.6% of the crystal, is defined with a methyl group carbon atom, C11, on atom C3 adjacent to the propyl-substituted C4 atom, and a methine group carbon atom, C6, adjacent to the pentyl-substituted C5 atom. A constitutional isomer (**3**), present as 47.4% of the crystal, is modeled with the methyl group carbon atom, C11, on atom C3 adjacent to the pentyl-substituted C4 atom, and a methine group carbon atom, C6, adjacent to the propyl-substituted C5 atom. Partially occupied water molecules are evident from the electron density maps, and near the methyl groups. The C3(methyl)-C6(H):C3(*H*)-C6(methyl) fragment occupancy was refined to a ratio of 0.704(8):0.296(8), and the C4(*n*-propyl):C4(*n*-pentyl) fragment occupancy ratio was 0.566(9):0.434(9). The molecules are packed in the crystal through hydrogen bonding with the amide hydrogen atoms and adjacent carbonyls. The intensity data was collected at experimental station 21-ID-D, Life Sciences-Collaborative Access Team (LS-CAT), at Advanced Photon Source, Argonne National Laboratory, and processed with XDS [7]. The structure was solved and refined with SHELX [8]. The final R-factor was 0.0799 for 1525 intensities greater than 2σ, and 0.0961 for all 2030 unique data. The X-ray analysis supports the proposed structures of **2** and **3** (Figure 4, Supplementary Figures S17 and S18). Therefore, **2** and **3** were determined as shown and named diazaquinomycin F and diazaquinomycin G, respectively. Crystallographic data for the structure of **2** and **3** were deposited under accession number CCDC 996646 and can be obtained free of charge from The Cambridge Crystallographic Data Centre. The ratio of **2** and **3** in the co-crystal does not necessarily reflect the weight percentage in the compound mixture, as evidenced by uneven H-6 resonances in the ^1^H-NMR spectrum (Supplementary Figures S11 and S14).

Structure numbered to emphasize DAQF occupancy in the co-crystal.

The known metabolite DAQA (**4**) was isolated and characterized based on comparison of ^1^H-NMR and HRMS data with those appearing in literature [5,9].

### 2.2. DAQA (**4**) Induces DNA Damage, Cell Cycle Arrest, and Apoptosis through Cleaved-PARP

Compounds **1** and **4** were tested for *in vitro* cytotoxicity against the ovarian cancer cell line OVCAR5. Dose response analysis of the isolated compounds revealed an LC_50_ of 9.0 μM for **1** and 8.8 μM for **4** after treatment of cells for 96 h (Supplementary Figure S1).

**Figure 5:**
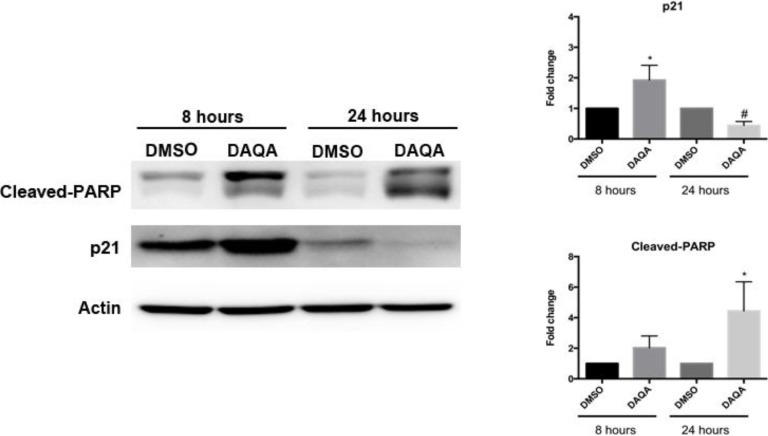
Compound **4** induces cell cycle arrest followed by apoptosis.

**Figure 6:**
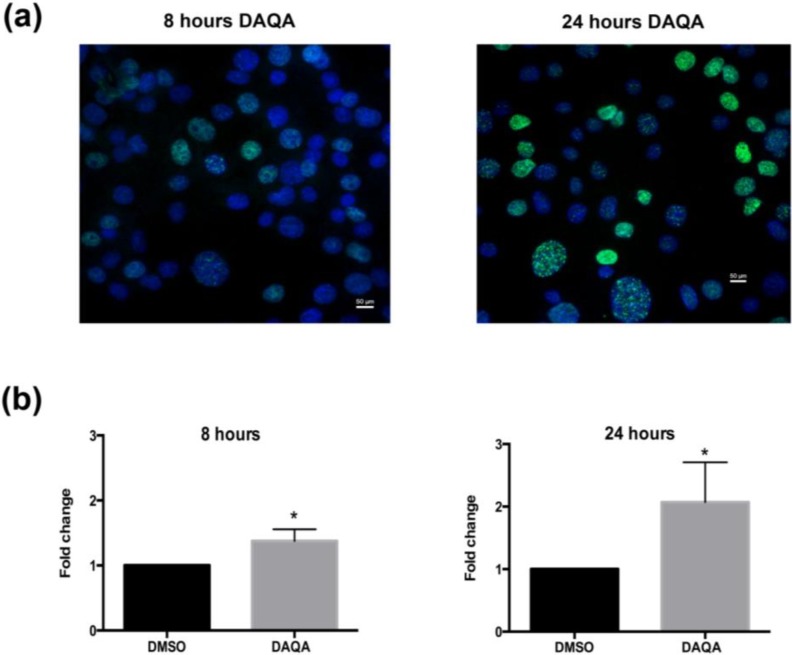
Compound **4** induces DNA damage in OVCAR5 cells. (**a**) H2A.X foci images taken after treatment of OVCAR5 cells with DAQA at 17.6 μM 8 h and 24 h; (**b**) Quantification of phospho-histone H2A.X foci as a fold increase over DMSO solvent control. Statistical significance is donated by * using student *t*-test. * *p* ≤ 0.05.

Further cell-based experiments were performed on **4** due to its high yield. Western blot analysis of OVCAR5 cells treated with 17.6 μM (LC_100_) of **4** showed increased levels of p21 (a cell cycle inhibitor) after 8 h (Figure 4). Interestingly, levels of p21 decreased after 24 h when compared to solvent control (Figure 4). Reduction of p21 protein after 24 h correlated with an increase in cleaved-PARP, an indication of apoptosis (Figure 5). The induction of cell cycle arrest, leading to apoptosis suggests significant DNA damage. To address this possibility, immunofluorescence for phospho-histone H2A.X was performed on OVCAR5 cells treated with **4** at 17.6 μM. Enhanced DNA damage, as monitored by increased phospho-histone H2A.X staining, was seen after 8 h and 24 h treatment with 17.6 μM of **4** when compared to solvent control (Figure 6a,b).

OVCAR5 cells treated with 17.6 μM of **4** showed induction of cell cycle arrest through increased p21 after 8 h, and induction of apoptosis through increased cleaved-PARP after 24 h. Densitometry of westerns was performed from three replicates. Statistical significance is donated by * for increased expression, and # for decreased expression. Student *t*-test was performed for all statistics and are represented as SEM +/−, * and # *p* ≤ 0.05.

Diazaquinomycins A and B were originally isolated from a *Streptomyces* sp. after exhibiting moderate inhibitory activity against four Gram-positive bacteria (three *Staphylococcus aureus* and one *Streptococcus faecium* IFO 3181 strains) in agar-based assays [5,9]. A follow-up publication by the same group reported that DAQA exhibited cytotoxicity against Vero and Raji cell lines, while also inhibiting thymidylate synthase in Ehrlich ascites carcinoma [10]. Additional studies were carried out in order to increase the solubility and bioactivity of DAQA, and some success was achieved through modification of the C-3 and C-6 methyl groups to short chain ester and ether derivatives [11]. Nearly 15 years later, DAQC was isolated from a *Streptomyces* sp.; this report was also the first mention of DAQD, though the metabolite was only observed through (−)-ESI MS experiments and was never fully characterized [6]. The observation of an aromatic resonance in the ^1^H spectrum (H-6) of **1** is a feature unique among existing diazaquinomycin analogs; previously reported structures of this class contain a methyl group at this position [5,6,9]. In the current study, though four secondary metabolites were isolated and characterized from strain F001, we observed ten additional diazaquinomycin analogs (based on the presence of characteristic UV spectra and MS^2^ fragmentation patterns) in LCMS data of our bioactive fractions. Positive ion values ranged from *m*/*z* 355.1 [M + H]^+^ to 425.2 [M + H]^+^, and given that UV spectra remained consistent among derivatives, structural modifications likely occurred in the α-substituted methyl and β-substituted alkyl groups. Finally, **1** and **4** exhibited moderate cytotoxicity toward OVCAR5 cells by inducing apoptosis and enhancing DNA damage.

## 3. Experimental Section

### 3.1. General Experimental Procedures

UV spectra were measured on a Shimadzu Pharma Spec UV-1700 spectrophotometer (Shimadzu, Kyoto, Japan). NMR spectra were obtained on a Bruker 600 MHz DRX NMR spectrometer (Bruker, Karlsruhe, Germany) equipped with an inverse 5 mm TXI cryogenic probe with *z*-axis pfg and XWINNMR version 3.5 operating software, and a 900 (226.2) MHz Bruker AVANCE NMR spectrometer (Bruker, Karlsruhe, Germany) equipped with an inverse 5 mm TCI cryogenic probe with *z*-axis pfg and TopSpin version 1.3 operating software at the University of Illinois at Chicago Center for Structural Biology (Chicago, IL, USA). Chemical shifts (δ) are given in ppm and coupling constants (*J*) are reported in Hz. ^1^H and ^13^C-NMR resonances of **1** and **2** are reported in Table 1. High-resolution mass spectra were obtained on a Shimadzu ion trap-time of flight (IT-TOF) spectrometer at the University of Illinois at Chicago Research Resources Center (Chicago, IL, USA). High-performance liquid chromatography (HPLC-UV) data were obtained using a Hewlett-Packard series 1100 system controller and pumps with a Model G1315A diode array detector (DAD) (Hewlett-Packard, Palo Alto, CA, USA) equipped with a reversed-phase C_18_ column (Phenomenex Luna, 100 × 4.6 mm, 5 μm) at a flow rate of 0.5 mL·min^−1^. Semi-preparative HPLC scale separations were performed using a Hewlett Packard Series 1050 system (Hewlett-Packard, Palo Alto, CA, USA) with a Phenomenex Luna semi-preparative C_18_ column (250 × 10 mm, 5 μm) at a flow rate of 2.4 mL·min^−1^. Preparative HPLC scale separations were performed using a Waters LC4000 System equipped with a Phenomenex Luna preparative C_18_ column (250 × 21.2 mm, 5 μm) at a flow rate of 16 mL·min^−1^.

### 3.2. Selection of Actinomycete Strain F001 for Further Investigation

We screened our library of *ca.* 2000 secondary metabolite fractions (from *ca.* 500 aquatic-derived actinomycete strains) in an *in vitro* single dose screen (20 μg/mL) against OVCAR5 and identified strain F001 as a promising bioactive lead, among other strains. Strain F001 (GenBank accession number KJ656126) shared 98% 16S rRNA gene sequence identity with the most closely related type strains *Streptomyces coacervatus* (GenBank accession number AB500703) [12], *Streptomyces hygroscopicus* subsp. *jinggangensis* (GenBank accession number NC_017765) [13], and *Streptomyces roseochromogenes* subsp. *oscitans* (GenBank accession number NZ_CM002285) [14].

### 3.3. Fermentation and Extraction

Strain F001 was cultured under two different media conditions. The culture that yielded **1** and **4** was grown in 44 × 1 L portions in Fernbach flasks containing high nutrient A1 medium (filtered ocean water, 10 g starch, 4 g yeast, 2 g peptone, 1 g calcium carbonate, 100 mg potassium bromide, and 40 mg iron sulfate) for 5 days at 21 °C while shaking at 220 rpm. The culture that yielded **2** and **3** was grown in 5 × 1 L portions in Fernbach flasks containing high nutrient CGS medium (filtered ocean water, 4 g casamino acids, 10 mL glycerol, and 5 g soy peptone) for 5 days at 21 °C while shaking at 220 rpm. Extraction methods for each growth condition were the same. Sterilized Amberlite XAD-16 resin (15 g·L^−1^) was added to each flask to absorb the extracellular secondary metabolites. The culture medium and resin were shaken for 8 h and filtered using cheesecloth to remove the resin. The resin, cell mass, and cheesecloth were extracted with acetone overnight, concentrated under vacuum, and partitioned between water and ethyl acetate. The organic layer of the A1 fermentation was dried under vacuum to afford 6.3 g of extract. The organic layer of the CGS fermentation was dried under vacuum to afford 1.1 g of extract.

### 3.4. Isolation and Characterization of Diazaquinomycins E (**1**), F (**2**), and G (**3**)

DAQE was isolated from the fermentation broth (A1 media) of strain F001. The organic layer from the liquid-liquid partition was fractionated using silica gel flash column chromatography (100 g of silica) eluting with an isocratic 95% chloroform (CHCl_3_):5% methanol (MeOH) solvent system to afford eight fractions. Using bioassay-guided fractionation, it was determined that fractions 2 and 3 contained the bioactive constituents, thus they were combined and separated using RP-C_18_ preparative HPLC (16 mL·min^−1^, gradient of 50% aqueous acetonitrile (ACN) to 100% ACN for 20 min, followed by an isocratic flow of 100% ACN for 10 min) to afford nine fractions. Fraction 8 (*t*_R_ 17.2 min, 14 mg) was separated using RP-C_18_ semi-preparative HPLC (2.4 mL·min^−1^, gradient of 50% aqueous ACN to 100% ACN for 25 min, followed by an isocratic flow of 100% ACN for 15 min) to afford diazaquinomycin E (**1**, *t*_R_ 22.1 min, 0.9 mg, 0.014% yield).

DAQF and DAQG were isolated from the fermentation broth (CGS media) of strain F001. The organic layer from the liquid-liquid partition of the culture extract was fractionated using silica gel flash column chromatography (100 g of silica) eluting with an isocratic 95% CHCl_3_:5% MeOH solvent system to afford eight fractions. Using bioassay-guided fractionation, it was determined that fraction 8 contained the bioactive constituents, thus, it was separated using normal phase silica gel (NP-Si) semi-preparative HPLC (2.0 mL·min^−1^, gradient of 99% CHCl_3_:1% MeOH to 90% CHCl_3_:10% MeOH for 10 min, followed by an isocratic flow of 90% CHCl_3_:10% MeOH for 15 min) to afford 7 fractions. Fraction 4 (*t*_R_ 18.1 min, 2.2 mg) was separated using NP-Si semi-preparative HPLC (2.0 mL·min^−1^, gradient of 99% CHCl_3_:1% MeOH to 90% CHCl_3_:10% MeOH for 10 min, followed by an isocratic flow of 90% CHCl_3_:10% MeOH for 10 min) to afford 7 fractions. Fraction 5 (*t*_R_ 19.3 min, 0.4 mg) was separated using NP-Si semi-preparative HPLC (2.0 mL·min^−1^, gradient of 99% CHCl_3_:1% MeOH to 90% CHCl_3_:10% MeOH for 10 min, followed by an isocratic flow of 90% CHCl_3_:10% MeOH for 10 min) to afford the co-eluting isomeric mixture of **2** and **3** (*t*_R_ 19.3 min, 0.28 mg, 0.025% yield).

**Diazaquinomycin E (1)**: Red solid (0.9 mg). UV (MeOH) λ_max_ (log ε) = 282.5 (3.90), 362.5 (3.24), and a broad peak with maximum at 486.0 (2.56) nm. ^1^H-NMR (900 MHz, CDCl_3_–1% CF_3_CO_2_D) and ^13^C-NMR (226.2 MHz, CDCl_3_–1% CF_3_CO_2_D), see Table 1. HRESI-ITTOF MS *m*/*z* 397.2198 [M + H]^+^ (calcd. for C_23_H_29_N_2_O_4_: 397.2127), *m*/*z* 395.1943 [M − H]^−^ (calcd. for C_23_H_27_N_2_O_4_: 395.1971), *m*/*z* 419.1997 [M + Na]^+^ (calcd. for C_23_H_28_N_2_O_4_Na: 419.1947), *m*/*z* 793.4211 [2M + H]^+^ (calcd. for C_46_H_57_N_4_O_8_: 793.4176), and *m*/*z* 815.3997 [2M + Na]^+^ (calcd. for C_46_H_56_N_4_O_8_Na: 815.3996).

**Diazaquinomycin F (2) and diazaquinomycin G (3)**: Red solid (0.28 mg). For UV, partial NMR, and HRMS data, see Supplementary Information (Table S1; Figures S11–S16).

### 3.5. OVCAR5 Cytotoxicity Assay

OVCAR5 cells were cultured in minimum essential media (Life Technologies, 11090-081, Carlsbad, CA, USA) and supplemented with 10% fetal bovine serum (Life Technologies, 16000-044) 1% l-glutamine (Life Technologies, 25030-081), 1% nonessential amino acids (Life Technologies, 11140-050), 1% sodium pyruvate (Life Technologies, 11360-050) and 1% penicillin/streptomycin (Life Technologies, 15140-122). OVCAR5 cells (5000/well) were plated in a 96 well plate one day prior to treatment. The next day, cells were treated with varying doses of the given compound in regular culture media. The doses tested were 10.0 μg/mL, 5.00 μg/mL, 2.50 μg/mL, 1.25 μg/mL, 0.625 μg/mL, 0.313 μg/mL, 0.156 μg/mL, and 0.0781 μg/mL. Doses higher than 10.0 μg/mL could not be tested due to compound insolubility in DMSO. Test plates were incubated at 37 °C with 5% CO_2_ for 96 h. After 96 h, media was removed from the cells and washed with cold PBS. Cells were permanently fixed to the culture plate using 5% trichloroactetic acid (TCA). A sulforhodamine B (SRB) assay was performed as previously reported [15]. Percent survival was calculated by comparing samples treated with **1** or **4**, and samples treated with the relevant volume of DMSO solvent control. Prism 6 GraphPad was used to graph the results and determine the LC_50_ in μM concentrations.

### 3.6. Western Blot Analysis

Cells were plated at a density of 50,000 cells in a 6 well plate one day before treatment. Cells were treated with 17.6 μM **4** for 8 h and 24 h. DMSO was used as a solvent control. Western blot gels were run as previously described [16]. An amount of 30 μg of cell lysate was run for each sample. p21 (#9247) and cleaved-PARP (#9541) from Cell Signaling were used to probe protein membranes at concentrations of 1:1000 in 5% milk/TBS-T. Actin (Sigma-Aldrich, St. Louis, MO, USA) was used as a loading control at a concentration of 1:1000. Anti-rabbit HRP-linked antibody (cell signaling) was used for all blots at 1:1000. Densitometry was performed using ImageJ software. All samples were performed in triplicate.

### 3.7. Immunofluorescence

Cells were plated at a density of 25,000 cells in a chamber slide (Millipore, PEZGS0816, Billerica, MA, USA) one day before treatment. After one day, cells were treated with 17.6 μM of **4** for 8 h and 24 h. After the treatment, cells were washed with 1X cold PBS and fixed with 4% paraformaldehyde, and permeabilized with 0.2% Triton-X100 in PBS for 10 min. Cells were then washed twice with 1× PBS and blocked with 10% goat serum in PBS. Phospho-histone H2A.X (Cell Signaling, #9178, Beverly, MA, USA) was incubated on the cells for 1 h at room temperature in 10% goat serum/PBS at a concentration of 1/100. After two PBS washes, cells were incubated with anti-rabbit AlexaFluor 488 (Life Technologies, Carlsbad, CA, USA) for 30 min at room temperature and mounted using Vectashield Mounting Medium with DAPI (Vector Laboratories, Burlingame, CA, USA). Images were taken on a Nikon E600 Microscope with a DS-Ri1 Digital Camera and NIS Elements Software (Nikon instruments, Melville, NY, USA). ImageJ software was used to count cells. The number of DAPI positive cells that were also positive for phosphor-histone H2A.X were expressed as a percentage of total DAPI stained cells. Only cells with defined foci were counted as positive. At least three random fields from three independent experiments were counted.

## 4. Conclusions

Three new diaza-anthracene analogs were identified, diazaquinomycin E (**1**), F (**2**), and G (**3**) from the culture broth of a marine-derived *Streptomyces* sp. DAQE and its known analog DAQA exhibited moderate cytotoxicity toward OVCAR5 cells (LC_50_ of 9.0 and 8.8 μM, respectively). At lethal concentrations of DAQA, evidence of DNA damage was seen with induction of apoptosis through cleaved-PARP. Among existing diazaquinomycins, **1**–**3** are the first reported members of the class to exhibit a variation in the diaza-anthracene core skeleton.

## Supplementary Files

Supplementary File 1 Supplementary Information (PDF, 1129 KB)
