# Supplementary material for: Diazaquinomycins E–G, Novel Diaza-Anthracene Analogs from a Marine-Derived Streptomyces sp
Source: Mar Drugs. 2014 Jun 11;12(6):3574–86. doi: 10.3390/md12063574 (PMC4071591; doi:10.3390/md12063574)
Supplement: Supplementary File 1 — Supplementary Information (PDF, 1129 KB) [file marinedrugs-12-03574-s001.pdf]

Supplementary Information

Figure S1. Dose response analysis of **1** and **3** in OVCAR5 cells.
Figure S2. ^{1}H-NMR spectrum (900 MHz) of diazaquinomycin E (**1**) in $CDCl_3$–1% CF_3CO_2D.
Figure S3. ^{13}C DEPTQ spectrum (226.2 MHz) of diazaquinomycin E (**1**) in $CDCl_3$–1% CF_3CO_2D.
Figure S4. COSY spectrum (600 MHz) of diazaquinomycin E (**1**) in $CDCl_3$–1% CF_3CO_2D.
Figure S5. HMBC spectrum (600 MHz) of diazaquinomycin E (**1**) in $CDCl_3$–1% CF_3CO_2D.
Figure S6. Selective 1D-TOCSY spectrum (600 MHz) of H_2-18 in diazaquinomycin E (**1**) in $CDCl_3$–1% CF_3CO_2D.
Figure S7. Selective 1D-TOCSY spectrum (600 MHz) of H_2-13 and H_2-14 in diazaquinomycin E (**1**) in $CDCl_3$–1% CF_3CO_2D.
Figure S8. Selective 1D-TOCSY spectrum (600 MHz) of H_2-20 in diazaquinomycin E (**1**) in $CDCl_3$–1% CF_3CO_2D.
Figure S9. Expanded HR-ESI-ITTOF mass spectrum of diazaquinomycin E (**1**).
Figure S10. UV spectrum of diazaquinomycin E (**1**) in ACN.
Table S1. ^{1}H and partial ^{13}C-NMR data of diazaquinomycin F (**2**) and diazaquinomycin G (**3**) in $CDCl_3$–1% CF_3CO_2D.
Figure S11. ^{1}H-NMR spectrum (600 MHz) of diazaquinomycin F (**2**) and diazaquinomycin G (**3**) in $CDCl_3$–1% CF_3CO_2D.
Figure S12. COSY spectrum (600 MHz) of diazaquinomycin F (**2**) and diazaquinomycin G (**3**) in $CDCl_3$–1% CF_3CO_2D.
Figure S13. HSQC spectrum (600 MHz) of diazaquinomycin F (**2**) and diazaquinomycin G (**3**) in $CDCl_3$–1% CF_3CO_2D.
Figure S14. HMBC spectrum (600 MHz) of diazaquinomycin F (**2**) and diazaquinomycin G (**3**) in $CDCl_3$–1% CF_3CO_2D.
Figure S15. Expanded HR-ESI-ITTOF mass spectrum of diazaquinomycin F (**2**) and diazaquinomycin G (**3**).
Figure S16. UV spectrum of diazaquinomycin F (**2**) and diazaquinomycin G (**3**) in ACN.
Figure S17. Co-crystal Structure of diazaquinomycin F (**2**) and diazaquinomycin G (**3**).
Figure S18. Deconvolution of co-crystal structure of diazaquinomycin F (**2**) and diazaquinomycin G (**3**).

Figure S1. Dose response analysis of **1** and **3** in OVCAR5 cells.

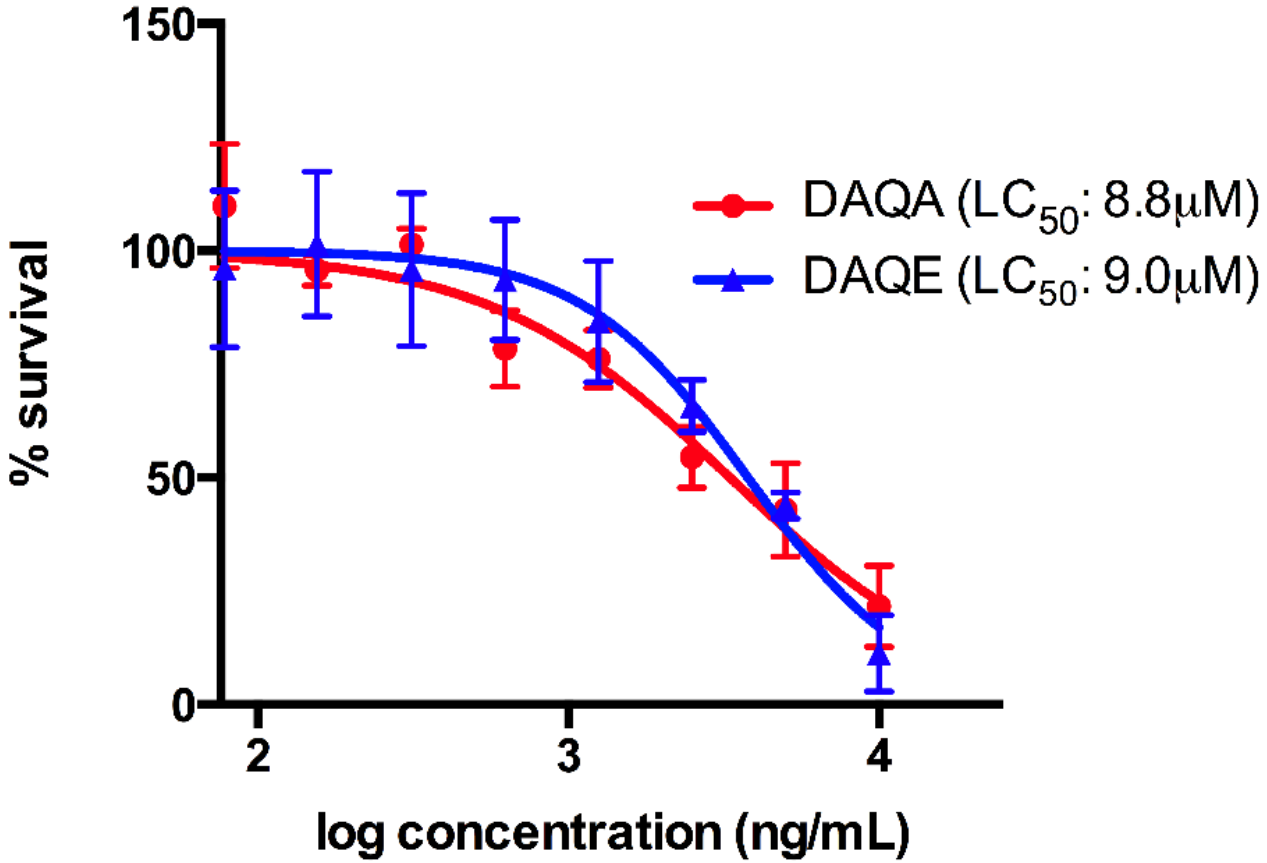

Concentrations represented as log of nanogram/mL. The LC_{50} value was determined using a non-linear curve fit on prism 6 GraphPad. These data represents average ± SEM from three replicates.

Figure S2. ^{1}H-NMR spectrum (900 MHz) of diazaquinomycin E (**1**) in $CDCl_3$–1% CF_3CO_2D.

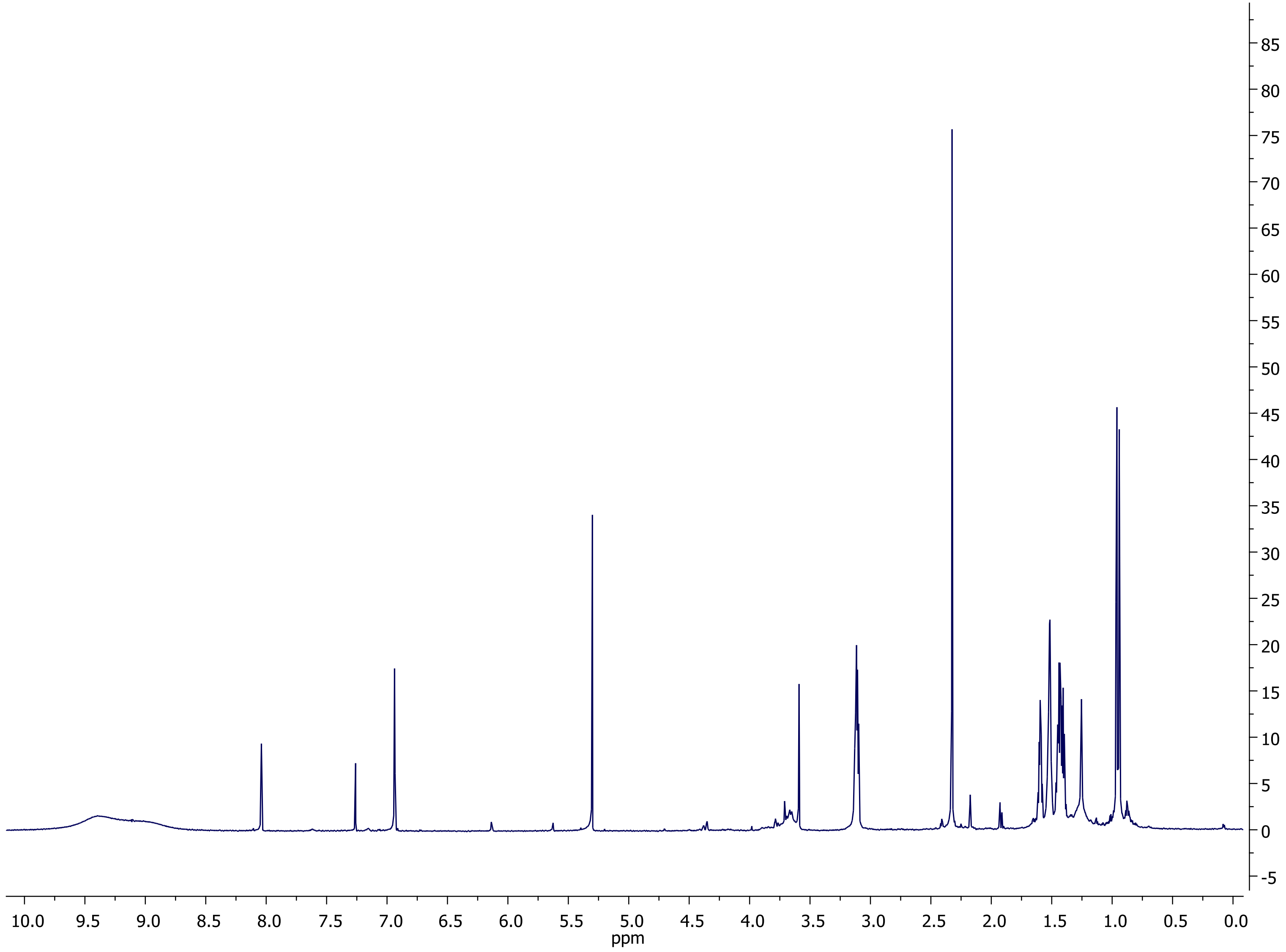

Figure S3. ^{13}C-DEPTQ spectrum (226.2 MHz) of diazaquinomycin E (**1**) in $CDCl_3$–1% CF_3CO_2D.

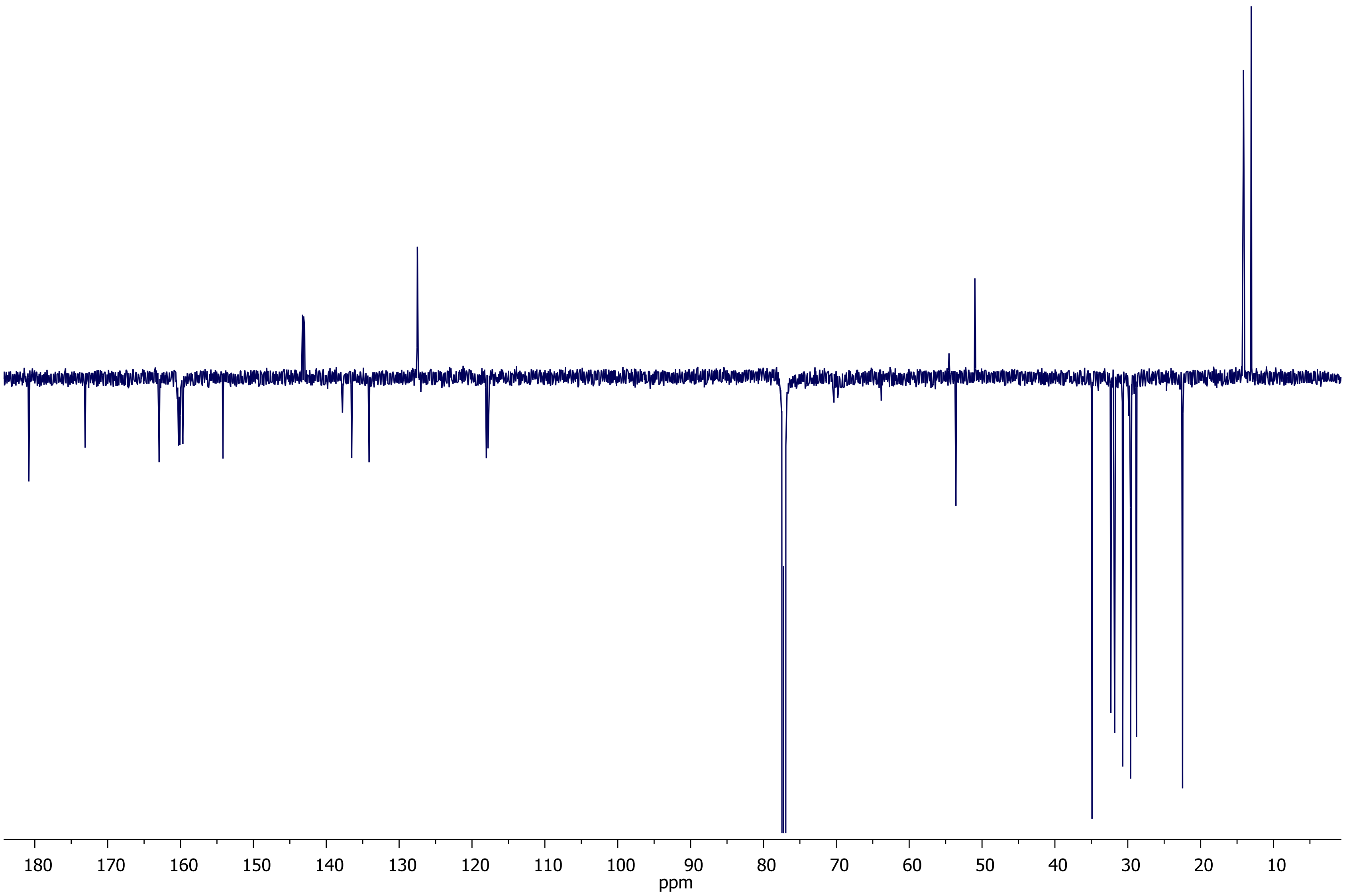

Figure S4. COSY spectrum (600 MHz) of diazaquinomycin E (**1**) in $CDCl_3$–1% CF_3CO_2D.

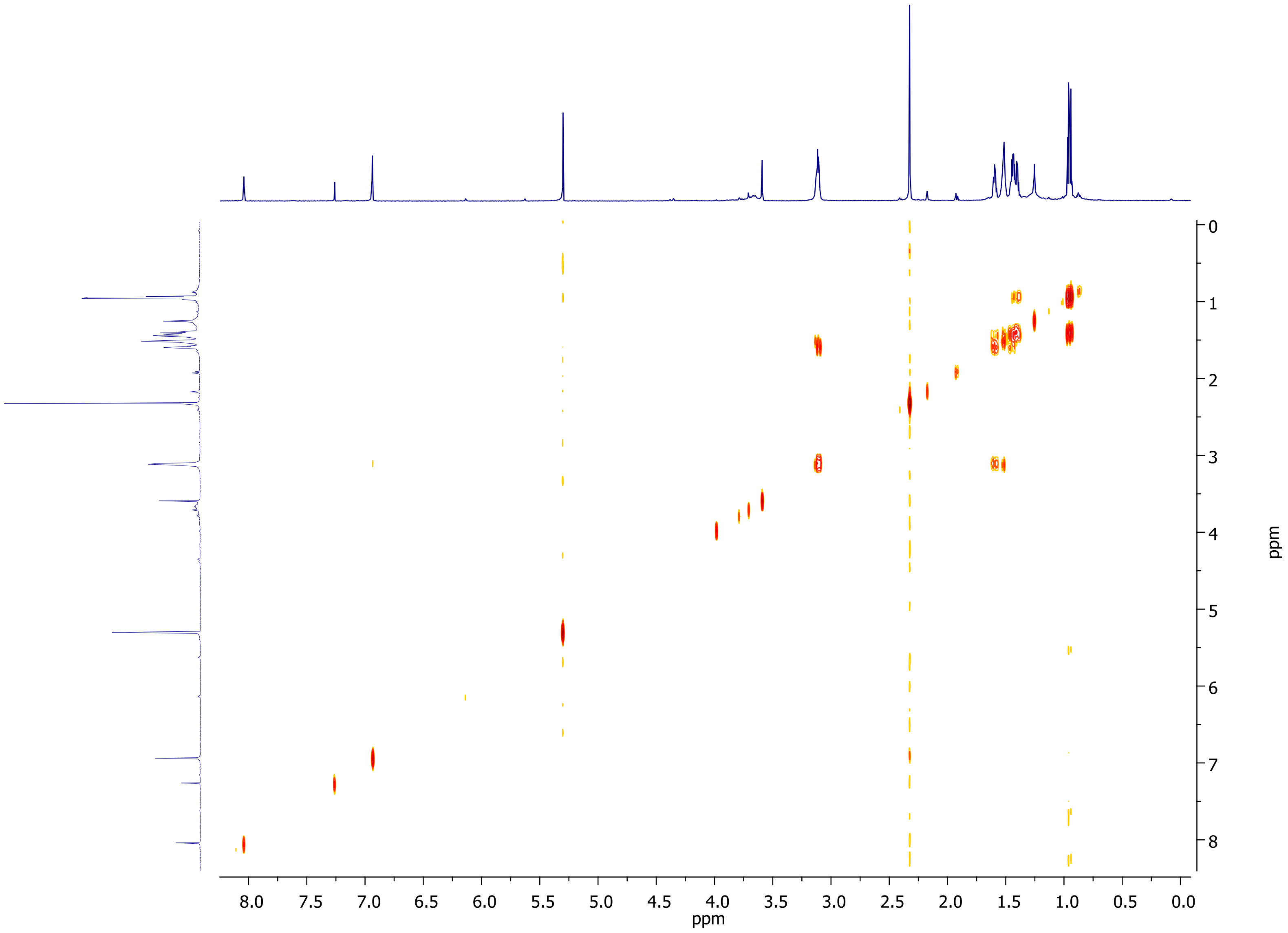

Figure S5. HMBC spectrum (600 MHz) of diazaquinomycin E (**1**) in $CDCl_3$–1% CF_3CO_2D.

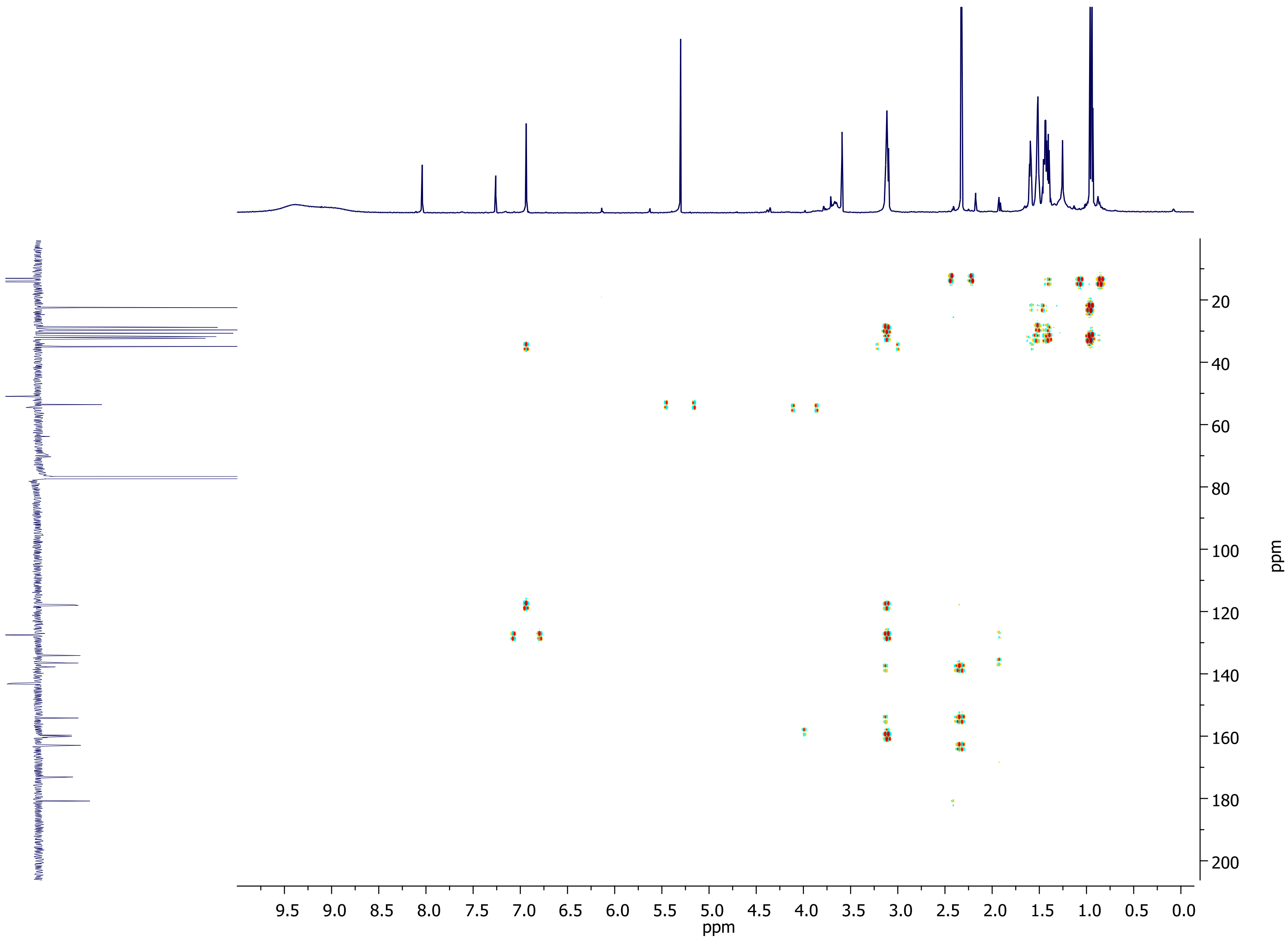

Figure S6. Selective 1D-TOCSY spectrum (600 MHz) of H_2-18 in diazaquinomycin E (**1**) in $CDCl_3$–1% CF_3CO_2D.

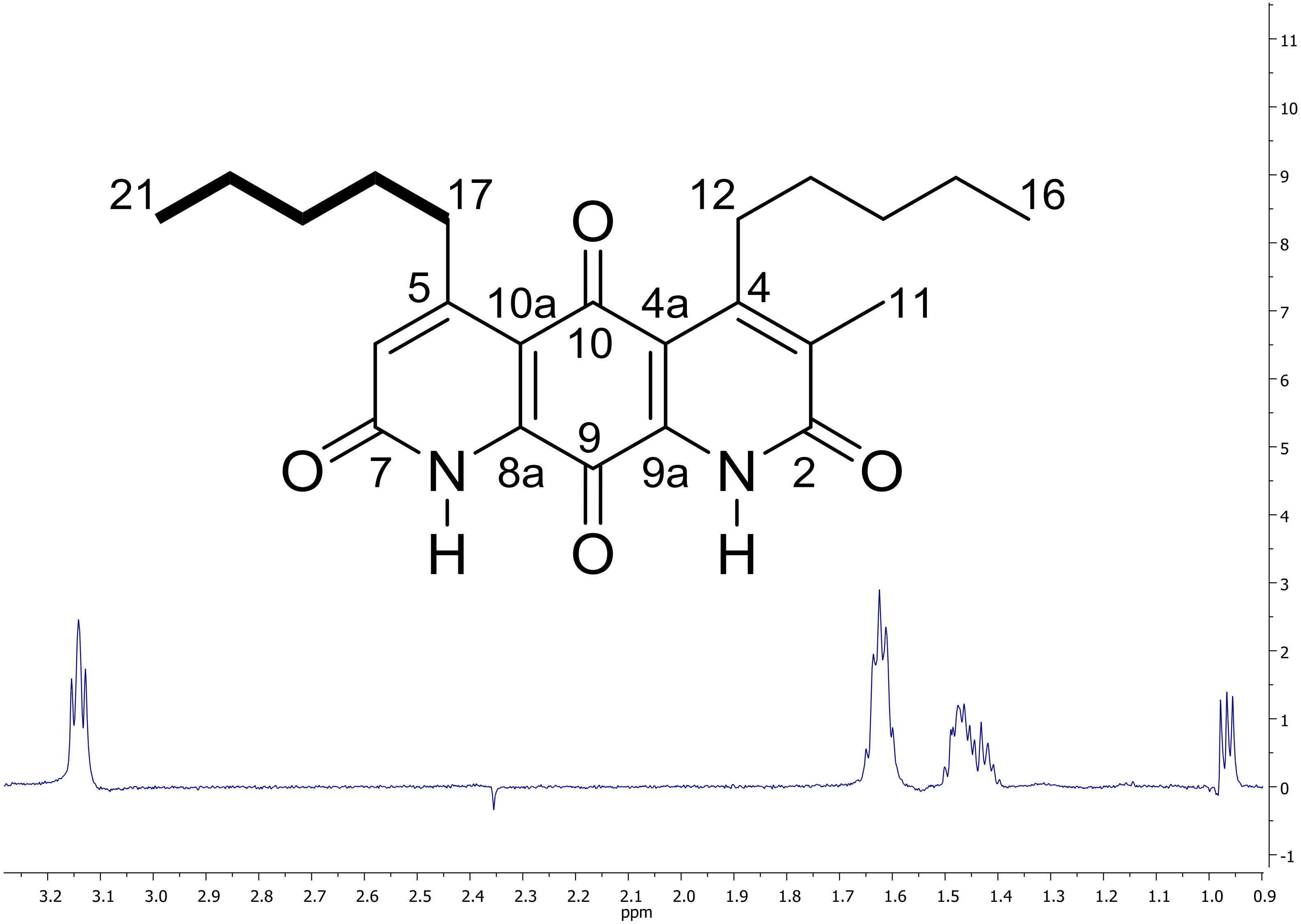

Figure S7. Selective 1D-TOCSY spectrum (600 MHz) of H_2-13 and H_2-14 in diazaquinomycin E (**1**) in $CDCl_3$–1% CF_3CO_2D.

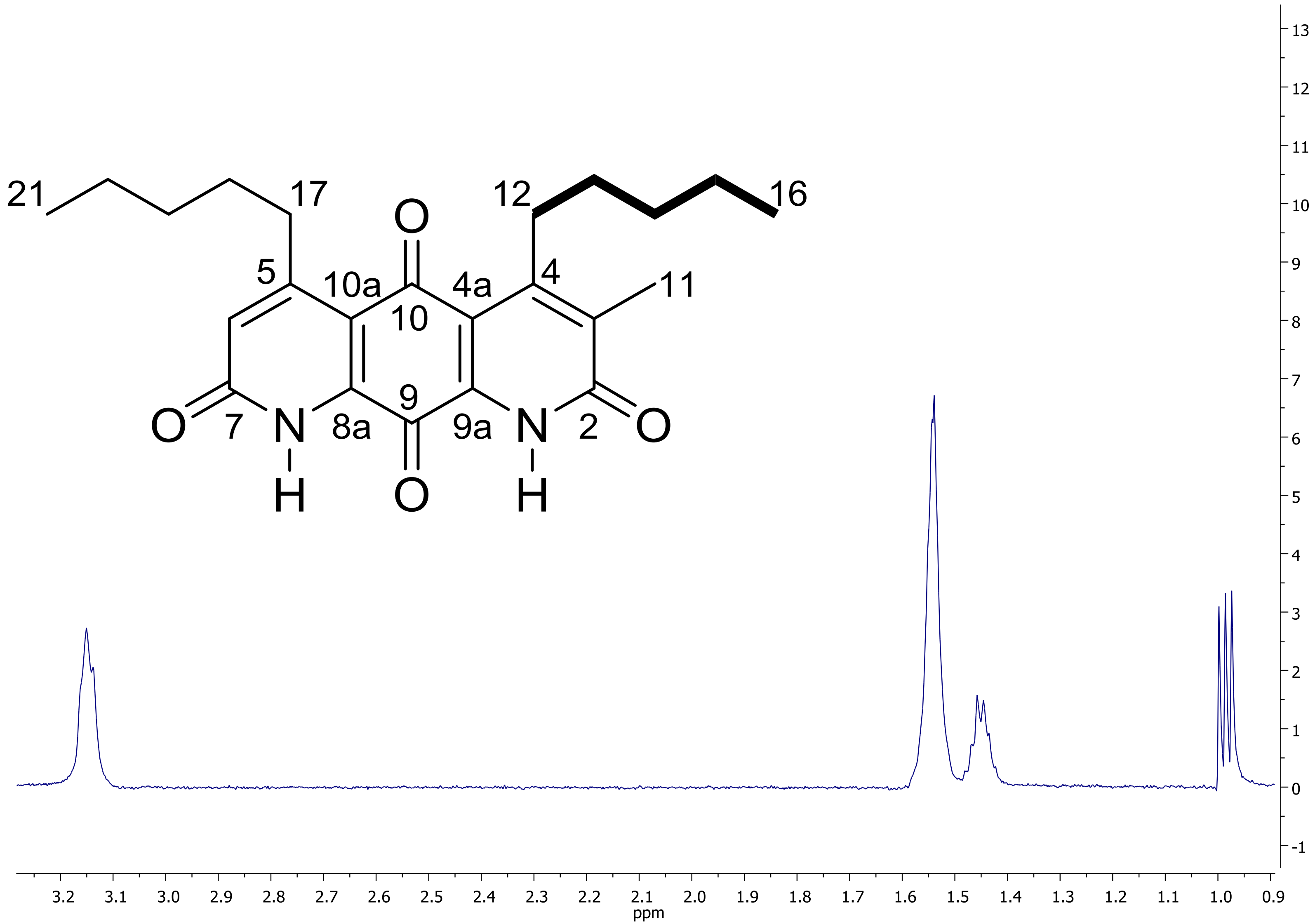

Figure S8. Selective 1D-TOCSY spectrum (600 MHz) of H_2-20 in diazaquinomycin E (**1**) in $CDCl_3$–1% CF_3CO_2D.

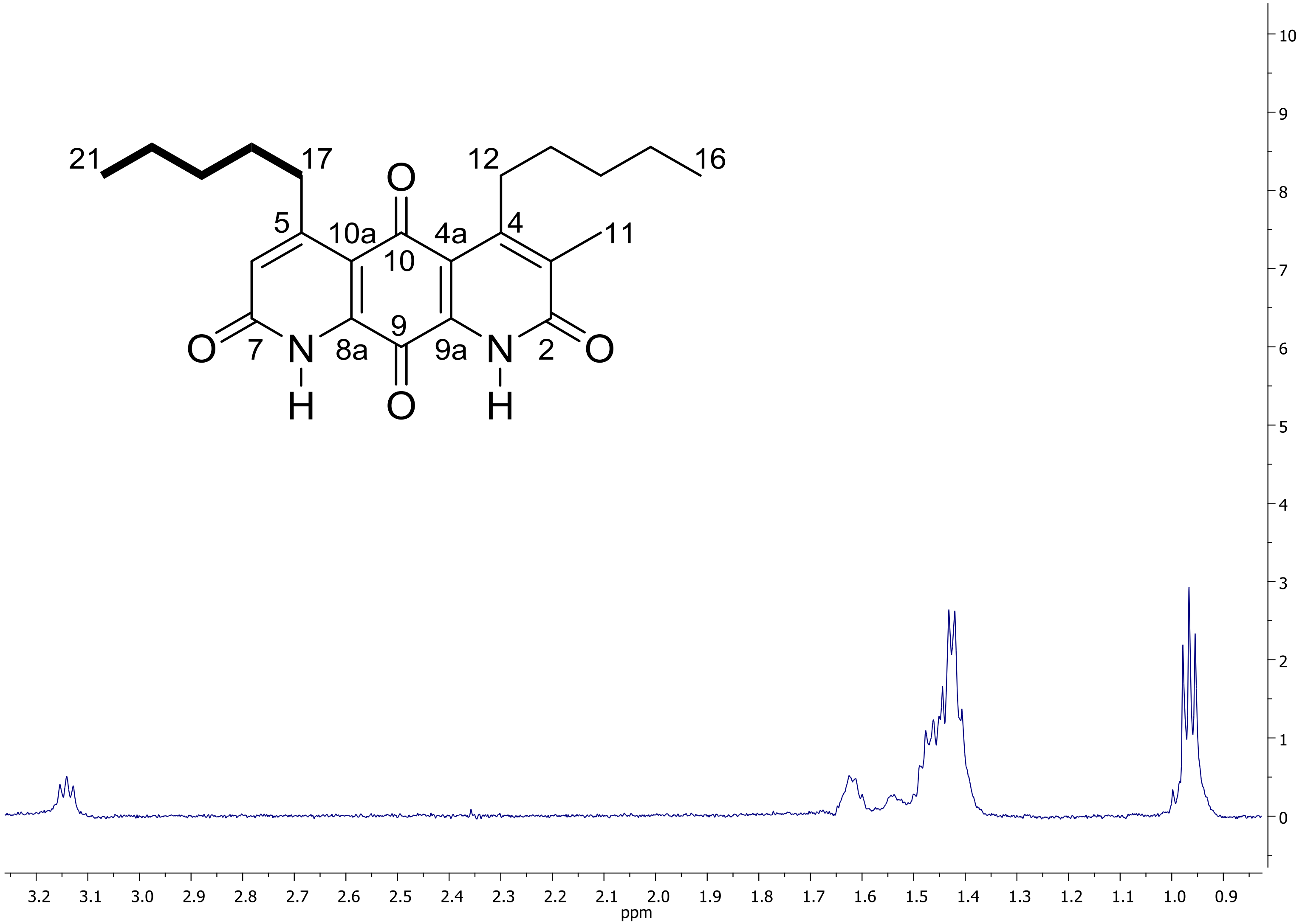

Figure S9. Expanded HR-ESI-ITTOF mass spectrum of diazaquinomycin E (**1**).

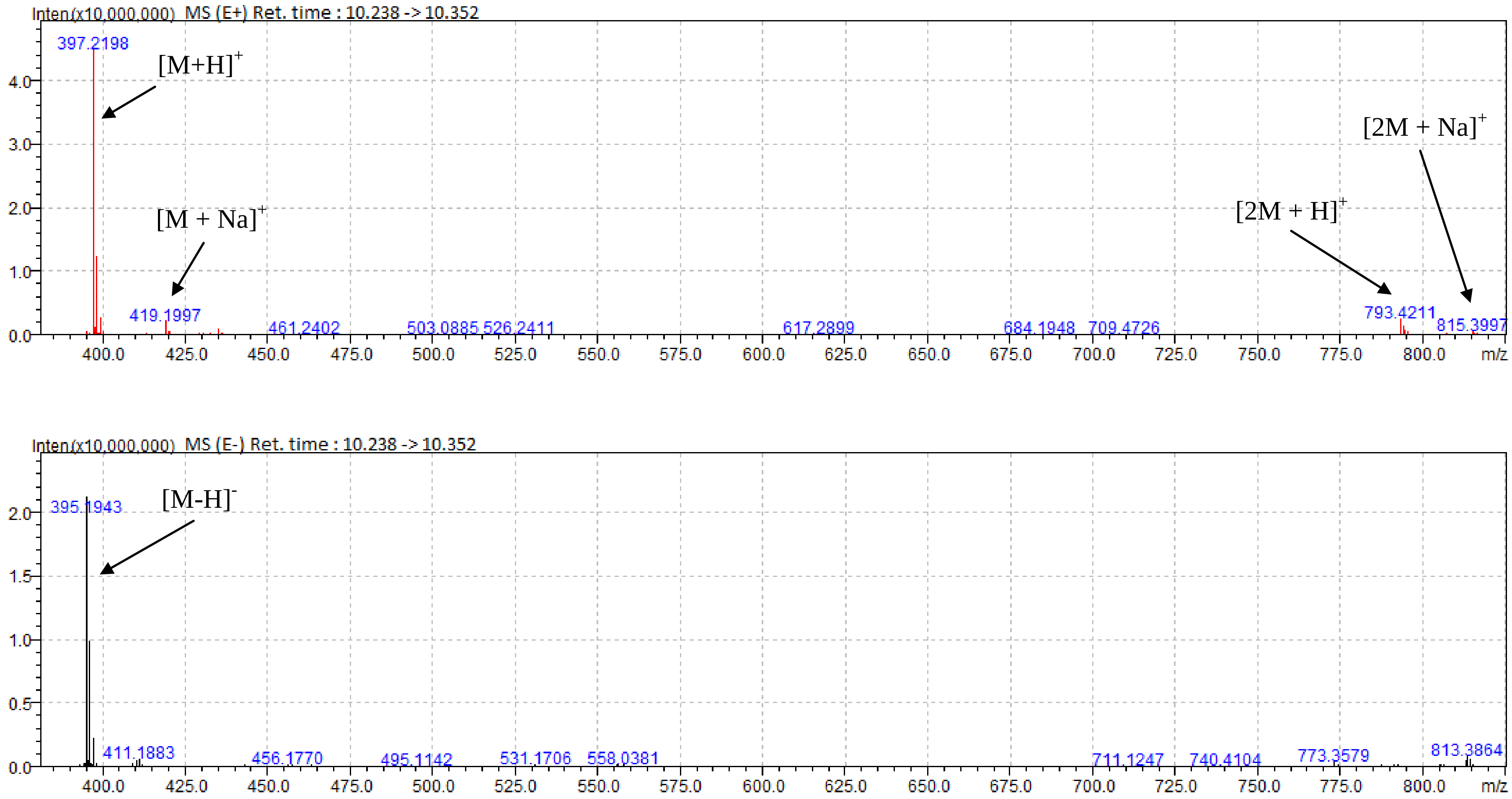

Figure S10. UV spectrum of diazaquinomycin E (**1**) in ACN.

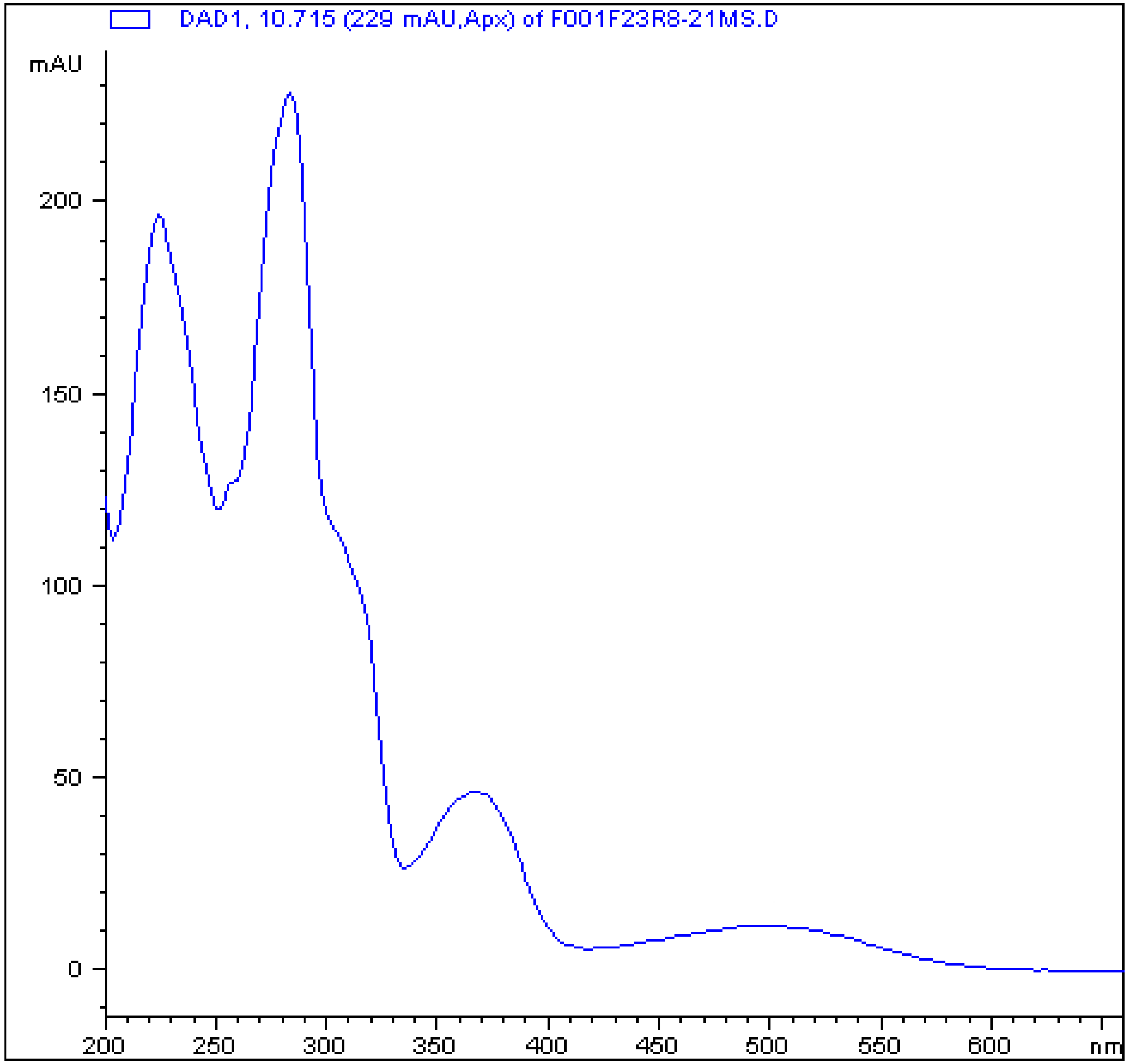

Table S1. ^{1}H and partial ^{13}C-NMR data of diazaquinomycin F (**2**) and diazaquinomycin G (**3**) in $CDCl_3$–1% CF_3CO_2D.

Position	^{13}C [a]	^{1}H mult. (*J*, Hz) [b]
2		
3		
4		
4a		
5		
6	127.2	6.95 s and 6.98 s [c]
7		
8a		
9		
9a		
10		
10a		
11	12.8	2.34 s
12	32.7	3.12 t (7.9)
13	22.3	1.57 m
14	14.4	1.14 t (7.3)
15	34.5	3.13 t (7.5)
16	29.3	1.59 m
17	31.4	1.44 m
18	22.2	1.39 m
19	13.7	0.94 t (7.0)
20		
21		

[a] Resonances extracted from HSQC data; [b] 600 MHz; [c] Chemically inequivalent α-methine hydrogens of DAQF and DAQG.

Figure S11. ^{1}H-NMR spectrum (600 MHz) of diazaquinomycin F (**2**) and diazaquinomycin G (**3**) in $CDCl_3$–1% CF_3CO_2D.

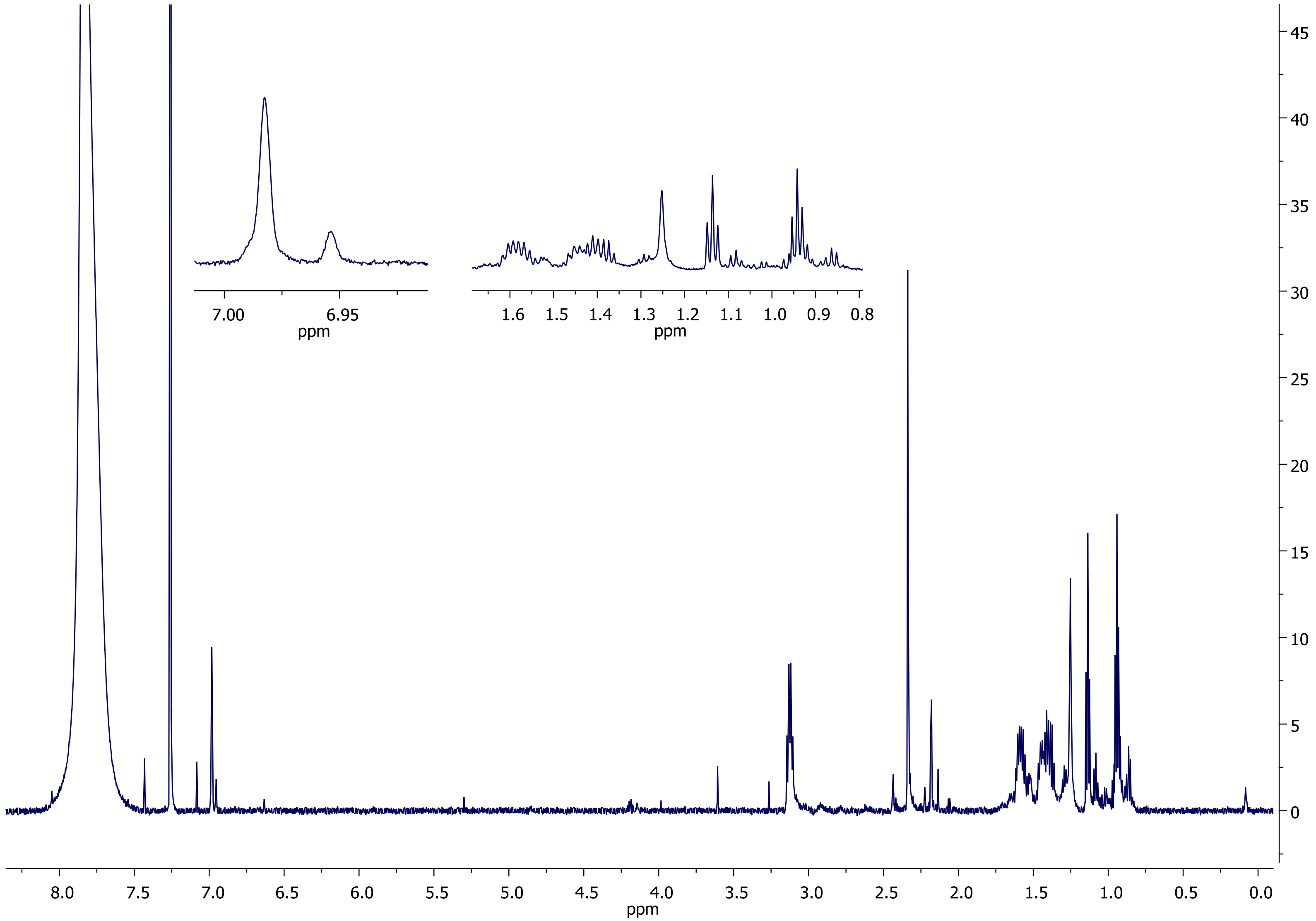

Figure S12. COSY spectrum (600 MHz) of diazaquinomycin F (**2**) and diazaquinomycin G (**3**) in $CDCl_3$–1% CF_3CO_2D.

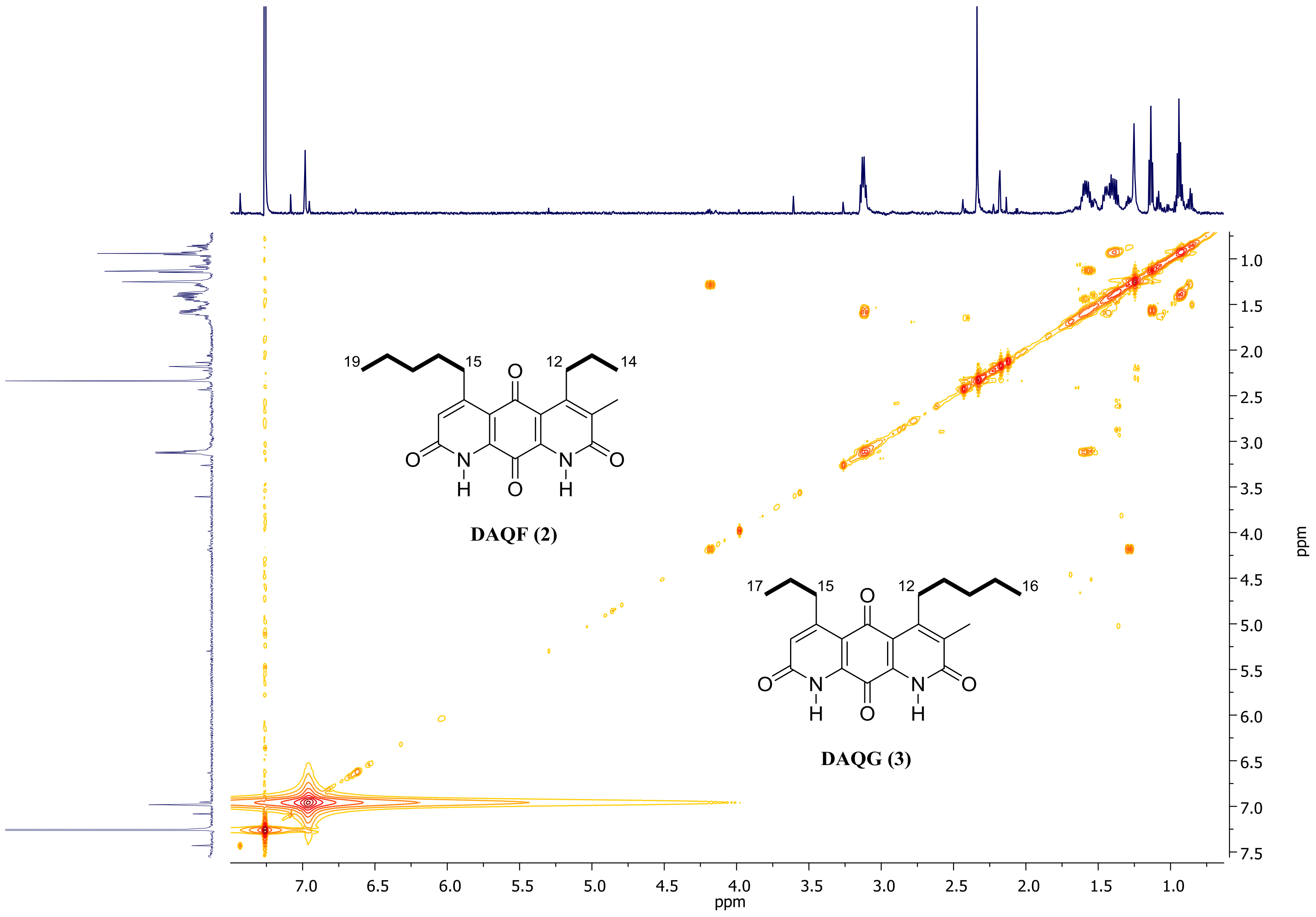

Figure S13. HSQC spectrum (600 MHz) of diazaquinomycin F (**2**) and diazaquinomycin G (**3**) in $CDCl_3$–1% CF_3CO_2D.

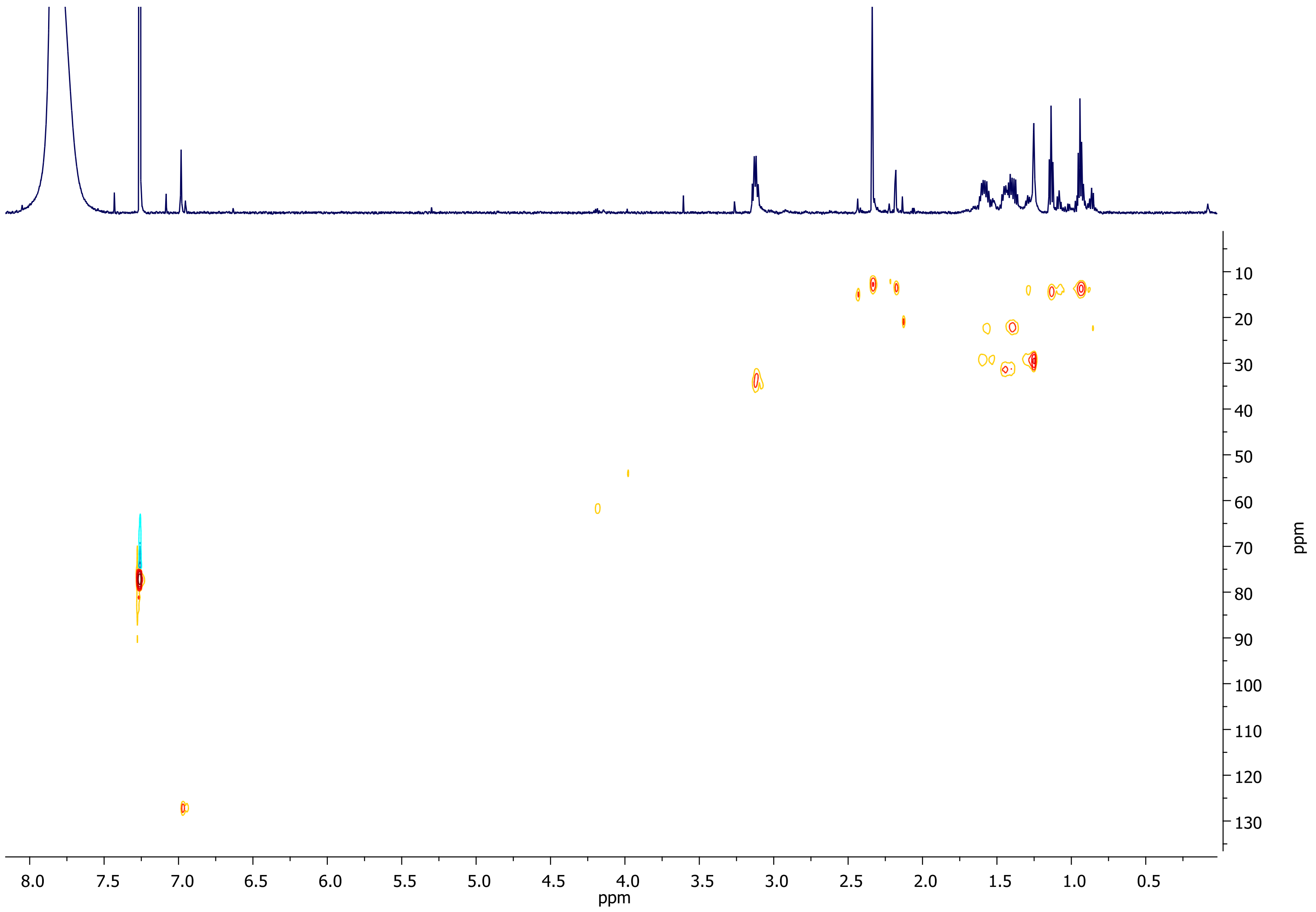

Figure S14. HMBC spectrum (600 MHz) of diazaquinomycin F (**2**) and diazaquinomycin G (**3**) in $CDCl_3$–1% CF_3CO_2D.

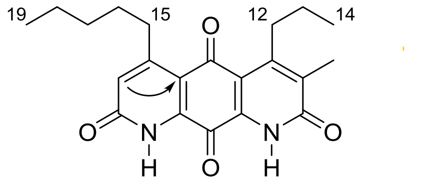

DAQF (2)

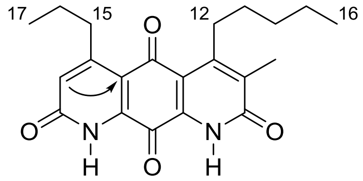

DAQG (3)

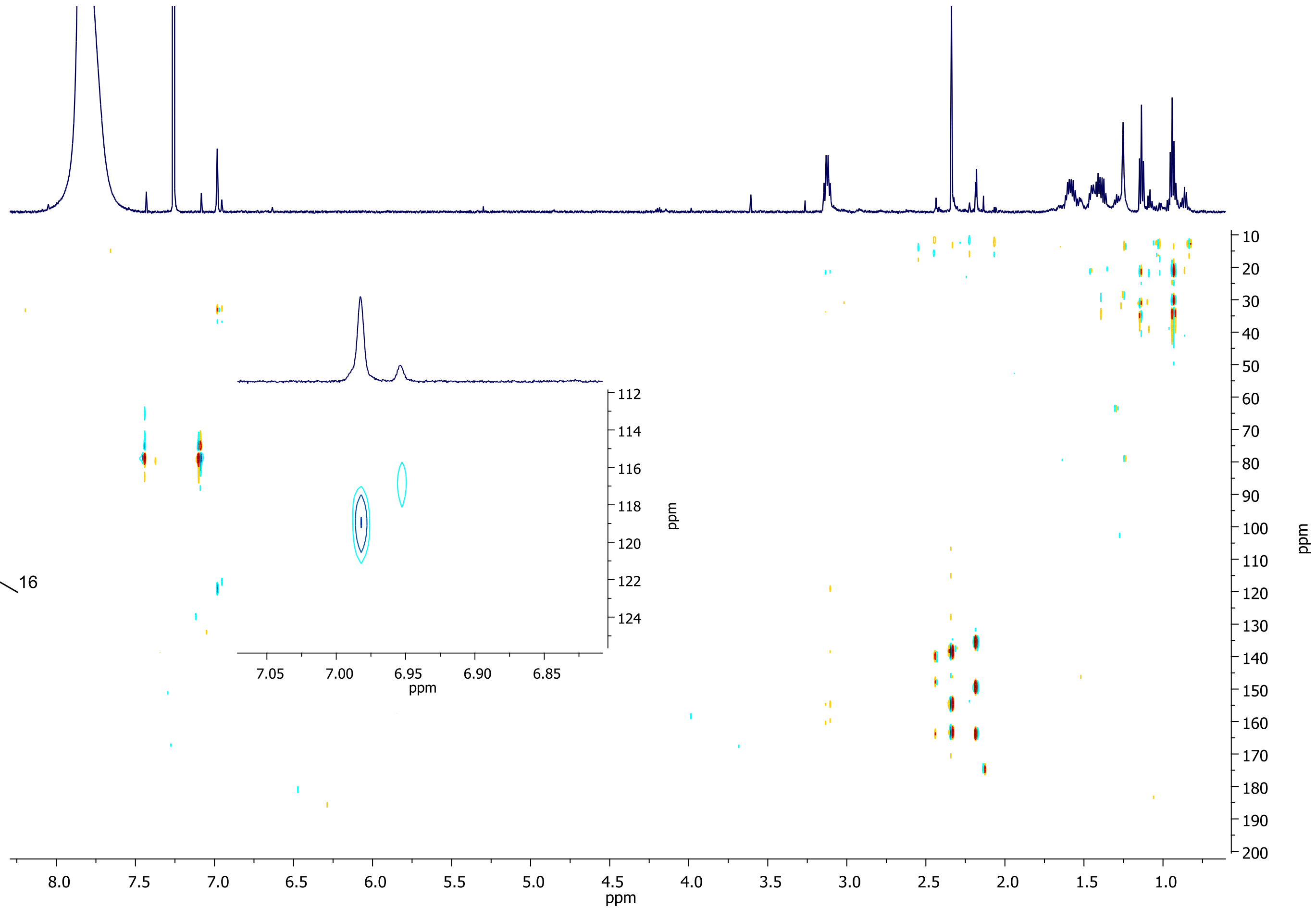

Inequivalency gives rise to two separate HMBC cross peaks (H6 to C10a in DAQF and DAQG).

Figure S15. Expanded HR-ESI-ITTOF mass spectrum of diazaquinomycin F (**2**) and diazaquinomycin G (**3**).

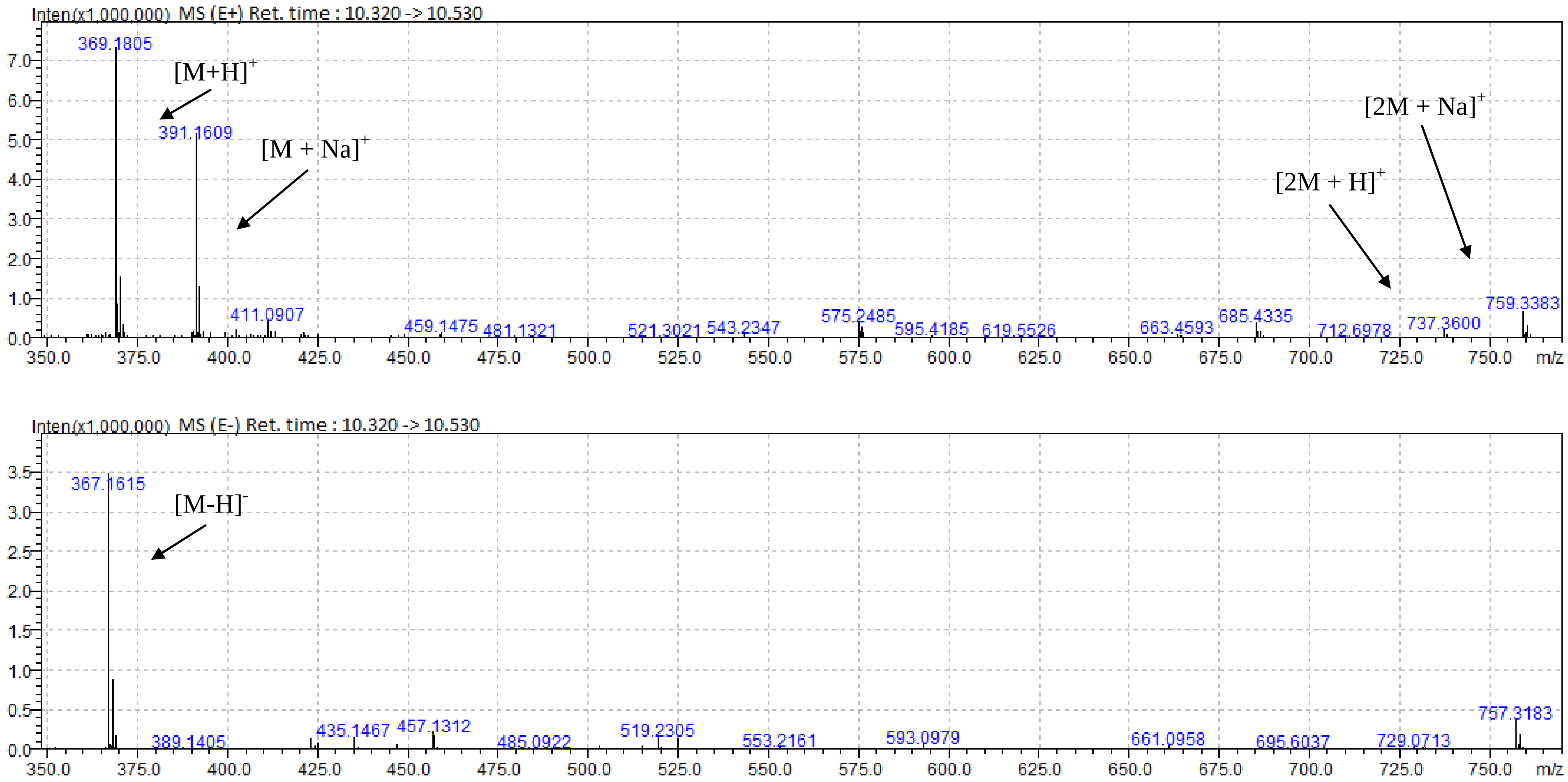

HRESI-ITTOF MS *m/z* 369.1805 [M + H]$^+$ (calcd. for $C_{21}H_{25}N_2O_4$: 369.1814), *m/z* 367.1615 [M − H]$^-$ (calcd. for $C_{21}H_{23}N_2O_4$: 367.1663), *m/z* 391.1609 [M + Na]$^+$ (calcd. for $C_{21}H_{24}N_2O_4Na$: 391.1634), *m/z* 737.3600 [2M + H]$^+$ (calcd. for $C_{42}H_{49}N_4O_8$: 737.3550), and *m/z* 759.3383 [2M + Na]$^+$ (calcd. for $C_{42}H_{48}N_4O_8Na$: 759.3370).

Figure S16. UV spectrum of diazaquinomycin F (**2**) and diazaquinomycin G (**3**) mixture in ACN.

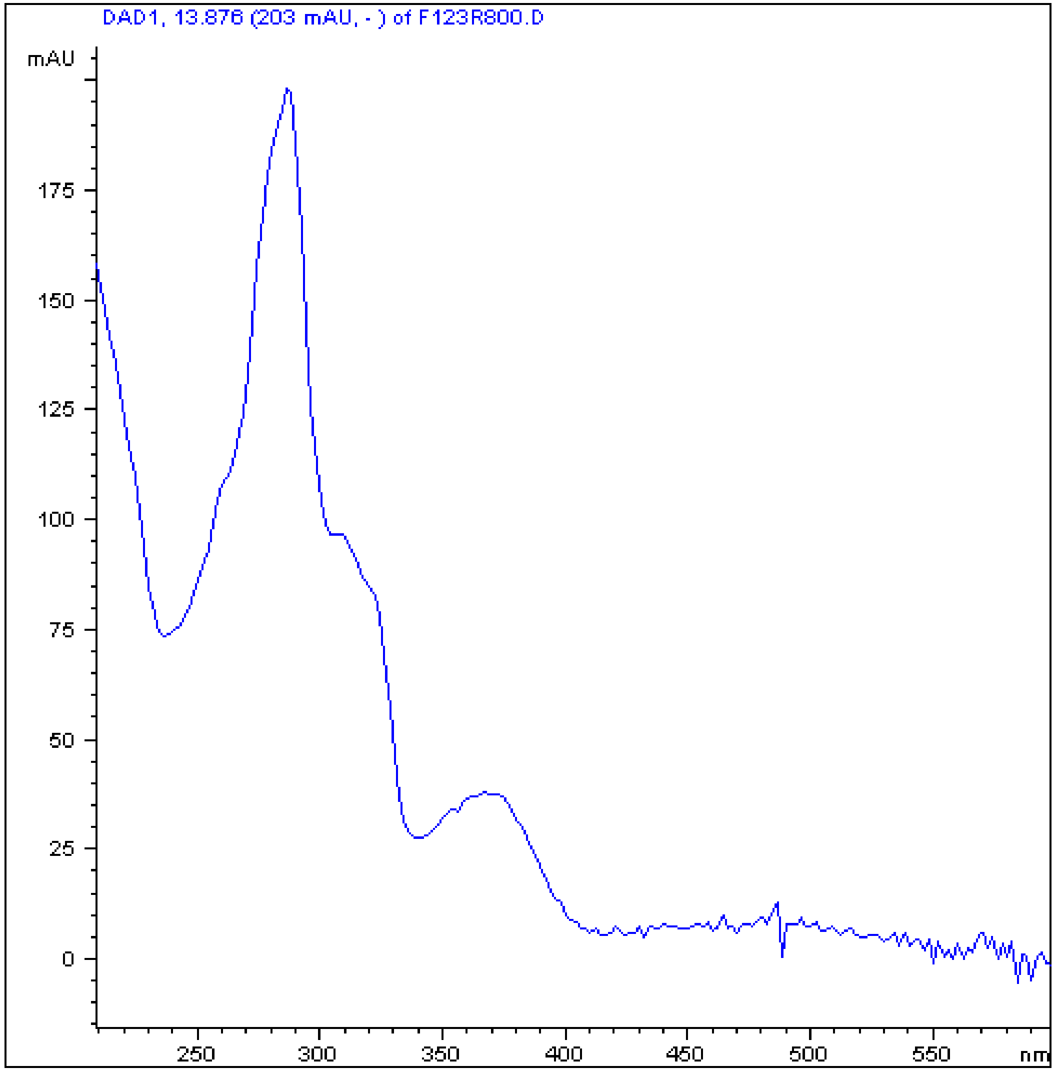

UV (MeOH) λ_{max} (log ε) = 280.5 (4.01), 359.0 (3.40), and a broad peak with maximum at 437.0 (2.66) nm.

Figure S17. Co-crystal Structure of diazaquinomycin F (**2**) and diazaquinomycin G (**3**).

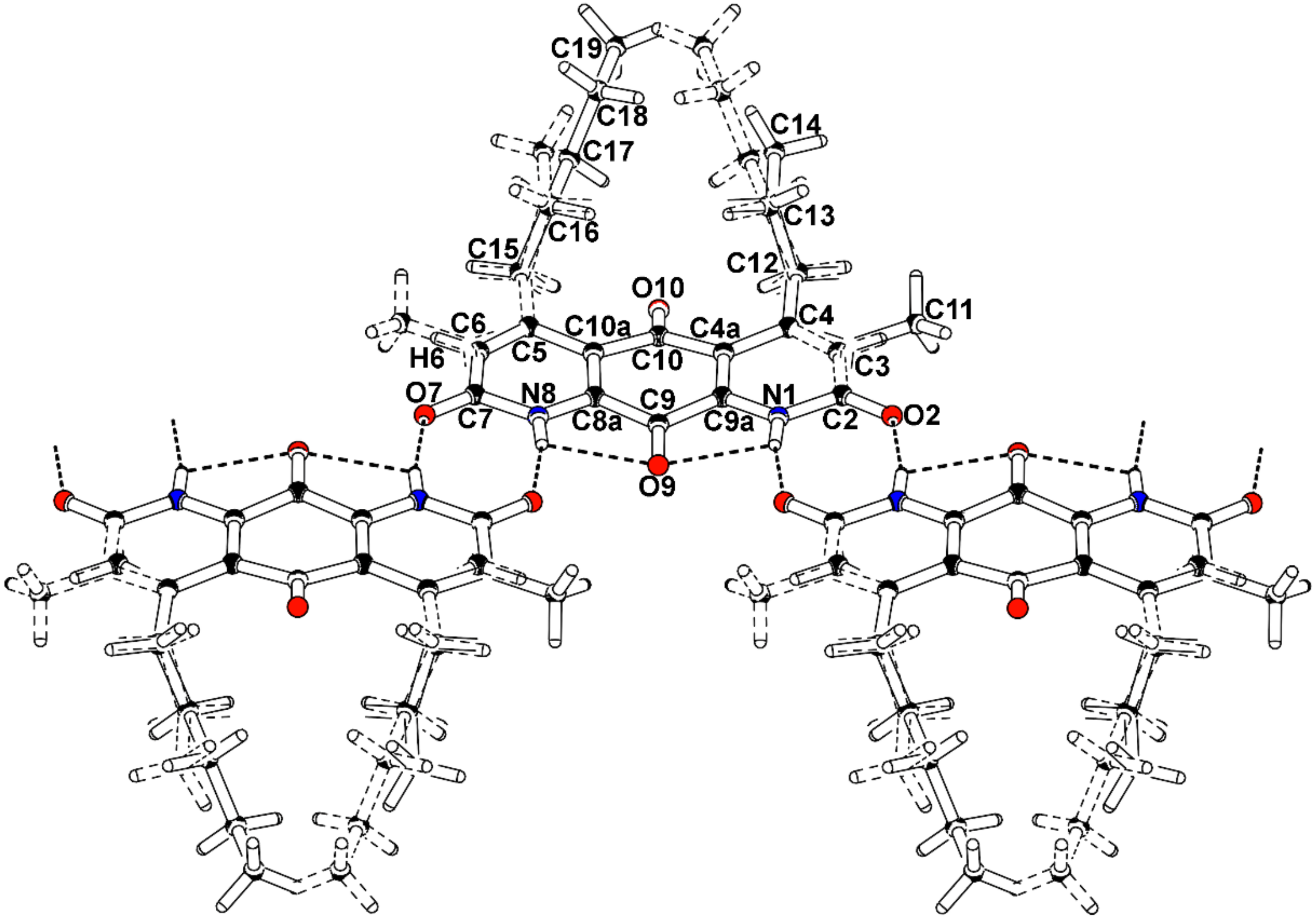

Structure numbered to emphasize DAQF occupancy in the co-crystal.

Figure S18. Deconvolution of co-crystal structure of diazaquinomycin F (**2**) and diazaquinomycin G (**3**).

A: 39.8% = (0.704 x 0.566) x 100

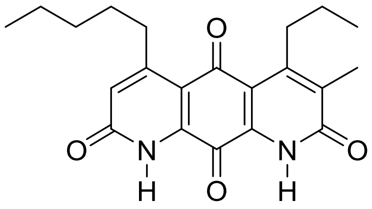

B: 30.6% = (0.704 x 0.434) x 100

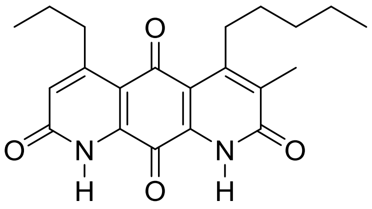

C: 16.8% = (0.296 x 0.566) x 100; same as **B**

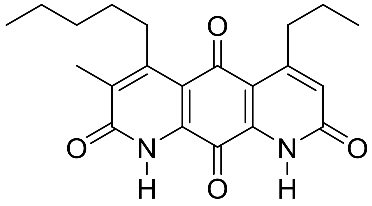

D: 12.8% = (0.296 x 0.434) x 100; same as **A**

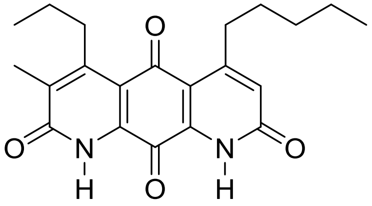

A + D = 39.8% + 12.8% = 52.6% DAQF

B + C = 30.6% + 16.8% = 47.4% DAQG

The methyl group at C3 is favored [0.704(8) to 0.296(8)], with the estimated standard deviation cited in parentheses; the propyl group at C4 is favored (0.566(9) to 0.434(9)). Taken together, DAQF resides at the crystallographic site 52.6% to DAQG (47.4%); As noted in the discussion, disordered water molecules are evident from the electron density maps. They occupy sites near the methine hydrogen atom, or near the end of the propyl group. If both disordered groups were equal, then they would each refine to 0.5.

© 2014 by the authors; licensee MDPI, Basel, Switzerland. This article is an open access article distributed under the terms and conditions of the Creative Commons Attribution license (http://creativecommons.org/licenses/by/3.0/).
